# Clonal structure and the specificity of vaccine-induced T cell response to SARS-CoV-2 Spike protein

**DOI:** 10.3389/fimmu.2024.1369436

**Published:** 2024-04-02

**Authors:** Saveliy A. Sheetikov, Alexandra A. Khmelevskaya, Ksenia V. Zornikova, Ivan V. Zvyagin, Alina S. Shomuradova, Yana V. Serdyuk, Naina T. Shakirova, Iuliia O. Peshkova, Aleksei Titov, Dmitrii S. Romaniuk, Irina A. Shagina, Dmitry M. Chudakov, Dmitry O. Kiryukhin, Olga V. Shcherbakova, Ekaterina G. Khamaganova, Vitalina Dzutseva, Andrei Afanasiev, Apollinariya V. Bogolyubova, Grigory A. Efimov

**Affiliations:** ^1^ Laboratory of Transplantation Immunology, National Medical Research Center for Hematology, Moscow, Russia; ^2^ Faculty of Biology, Lomonosov Moscow State University, Moscow, Russia; ^3^ Center for Precision Genome Editing and Genetic Technologies for Biomedicine, Institute of Translational Medicine, Pirogov Russian National Research Medical University, Moscow, Russia; ^4^ Genomics of Adaptive Immunity Department, Shemyakin-Ovchinnikov Institute of Bioorganic Chemistry, Moscow, Russia; ^5^ Central European Institute of Technology, Masaryk University, Brno, Czechia; ^6^ Novosibirsk State University, Medical School, Novosibirsk, Russia; ^7^ NPO Petrovax Pharm LLC, Moscow, Russia

**Keywords:** T cell, vaccination, SARS-CoV-2, adenoviral vaccine, T cell receptor, spike protein, TCR sequencing

## Abstract

Adenovirus vaccines, particularly the COVID-19 Ad5-nCoV adenovirus vaccine, have emerged as promising tools in the fight against infectious diseases. In this study, we investigated the structure of the T cell response to the Spike protein of the SARS-CoV-2 virus used in the COVID-19 Ad5-nCoV adenoviral vaccine in a phase 3 clinical trial (NCT04540419). In 69 participants, we collected peripheral blood samples at four time points after vaccination or placebo injection. Sequencing of T cell receptor repertoires from Spike-stimulated T cell cultures at day 14 from 17 vaccinated revealed a more diverse CD4^+^ T cell repertoire compared to CD8^+^. Nevertheless, CD8^+^ clonotypes accounted for more than half of the Spike-specific repertoire. Our longitudinal analysis showed a peak T cell response at day 14, followed by a decline until month 6. Remarkably, multiple T cell clonotypes persisted for at least 6 months after vaccination, as demonstrated by ex vivo stimulation. Examination of CDR3 regions revealed homologous sequences in both CD4^+^ and CD8^+^ clonotypes, with major CD8^+^ clonotypes sharing high similarity with annotated sequences specific for the NYNYLYRLF peptide, suggesting potential immunodominance. In conclusion, our study demonstrates the immunogenicity of the Ad5-nCoV adenoviral vaccine and highlights its ability to induce robust and durable T cell responses. These findings provide valuable insight into the efficacy of the vaccine against COVID-19 and provide critical information for ongoing efforts to control infectious diseases.

## 1 Introduction

The COVID-19 pandemic has prompted the international scientific community and pharmaceutical companies to develop numerous vaccine candidates and accelerate clinical trials. As a result, a number of these vaccines have received emergency or full approvals from national regulatory authorities in various countries. The use of novel vaccine platforms, such as adenoviral and RNA vaccines, has become widespread and widely accepted. Several studies have shown that these vaccines can induce effective antibody and T cell responses and provide protection against the wild-type virus (1–5).

Cellular immune responses may be important in the control and elimination of primary SARS-CoV-2 infection. Studies in acute and convalescent COVID-19 patients have observed an association between SARS-CoV-2-specific T cell responses and milder disease (6–9). Rapid activation of cytotoxic T cells has been shown to correlate with rapid viral clearance and mild disease, and its kinetics are synchronized with the development of the humoral response (10–12). In particular, it was observed that individuals with a pre-existing cross-reactive robust T cell response also had high antibody titers and a lower incidence of COVID-19 infection during follow-up compared to those with a weak cellular and humoral response (13). In addition, an effective T cell response alone, even without seroconversion, was shown to be sufficient to control infection (13–15). Approximately 74% of patients with lymphoid malignancies who have a severely compromised humoral immune response demonstrated a detectable T cell response (16, 17). This suggests that T cells are a sufficient protective mechanism in immunocompromised patients.

In contrast to neutralizing antibodies, T cells have been shown to recognize both the Spike protein responsible for facilitating viral entry into host cells, as well as epitopes from other proteins (6, 18–23). Multiple studies indicate that nucleocapsid and membrane proteins are among the most immunodominant targets (19, 20, 24). Targeted approaches determine the epitope specificity, HLA restriction, and clonality of the T cell responses in recovered patients (21, 23, 25–27). Interestingly, the average T cell response of a recovered individual recognizes only a handful of immunodominant epitopes and occupies less than 0.5% of the total T cell receptor (TCR) repertoire of peripheral memory T cells after infection (20, 28). The magnitude of the T cell response decreases over time differently depending on the immunodominance of the epitope. Initial richness of clonal diversity is critical for prolonged persistence of epitope-specific T cells (29, 30).

Even in individuals with a strong, broad and sustained T cell response, the emergence of new strains of SARS-CoV-2 poses a challenge to the immune system. The virus evades the immune response by mutating immunodominant regions of proteins. While it was expected that mutations in Spike protein could lead to complete evasion humoral immunity, studies show reduced, yet retained, antibody recognition of the virus mutants (31–33). Like B cell immunogenic regions, T cell epitopes can undergo mutations, potentially allowing new strains to evade T cell recognition and increase infectivity and mortality (34–36). This underscores the importance of monitoring the evolution of new strains and designing future vaccines accordingly. Despite these concerns, recognition of even a single epitope has been shown to effectively control the virus and rapidly terminate infection (37, 38). In addition, recognition of the SARS-CoV-2 epitope may be mediated by cross-reactive T cells specific for common cold coronaviruses (39).

During the pandemic, adenoviral and RNA-based vaccines have proven to be effective platforms for the rapid application of genetic engineering techniques to immunize against novel pathogens. Currently, the most commonly applied vaccines employ the Spike protein encoded within mRNA lipoparticles, adenoviral vectors, inactivated vaccines, or subunit recombinant vaccines with adjuvants because of Spike immunogenicity and ability to induce the production of virus-neutralizing antibodies in SARS-CoV-2-infected individuals (2–6, 18, 19, 40–42). Studies have shown that the amplitude of the Spike-specific T cell-mediated immune response can vary depending on the type of vaccine used. A comparative analysis between one of the mRNA vaccines (BNT162b2) and one of the recombinant adenoviral vaccines (ChAdOx1) showed that T cell activation efficiency was 1.4 times higher with the use of adenoviral vectors, while mRNA vaccines showed antibody titers that were 2.9 times higher (43, 44). However, little is known about the antigen-specific T cell repertoire generated after vaccination with an adenoviral vector. Recognition of immunodominant epitopes in vaccinated individuals and the dynamics of this process remain poorly understood.

To address this knowledge gap, the current study focuses on the SARS-CoV-2 Spike protein as a model antigen incorporated into an adenoviral vaccine that was widely used to vaccinate healthy individuals during the pandemic. We performed a longitudinal analysis of Spike-specific T cells in volunteers who received a single-component Ad5-nCoV vaccine. Using an immunosequencing approach, we obtained sequences of T cell receptors and monitored the presence of these clonotypes in peripheral blood up to 6 months after vaccination. We found that a polyclonal T cell response is generated after vaccination and is detectable up to 6 months after vaccination. We also identified differences in the number and size of CD4^+^ and CD8^+^ clonotypes and confirmed recognition of one of the immunodominant epitopes from the Spike protein. Our results contribute to the understanding of the immunogenicity of adenovirus vaccines. They are likely to be extrapolated to antigens from other pathogens - candidates for future adenovirus vaccines.

## 2 Results

### 2.1 Measurement of the Ad5-nCoV vaccine-induced T cell response

**Figure 1:**
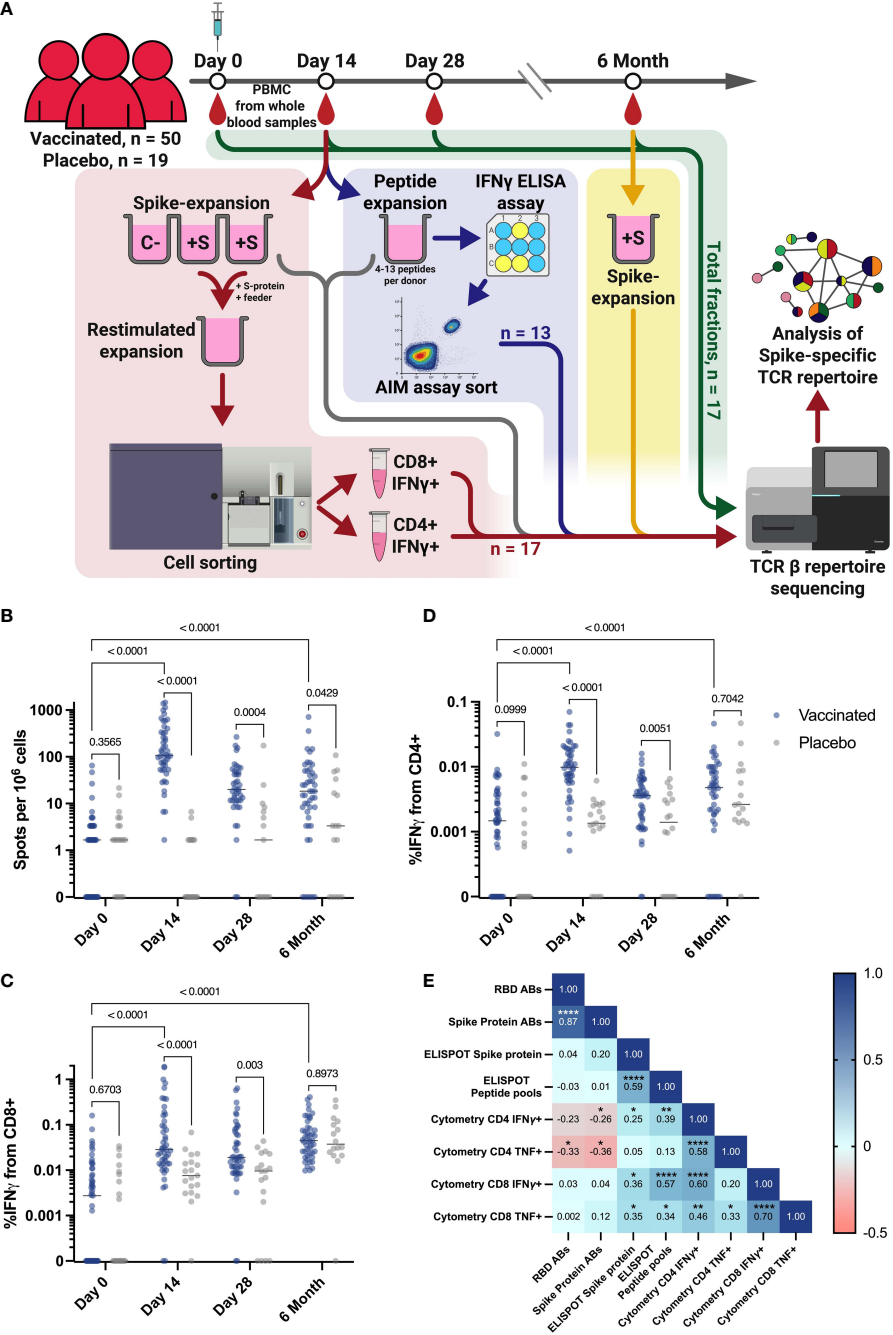
Study workflow and T cell response in vaccination and placebo groups measured by ELISPOT and flow cytometry. **(A)** Workflow for processing samples received from clinical trial participants. Blood samples collected from each individual at each time point were assessed for T cell responses by ELISPOT and intracellular cytokine staining followed by flow cytometry. Workflows for T cell expansion followed by IFNγ^+^ sorting, sequencing of total PBMC fractions, and peptide-specific expansion followed by IFNγ ELISA (enzyme-linked immunosorbent assay) and further activation assay (AIM assay) cell sorting are shown in red, green, and blue, respectively. Fractions of expanded cultures that were also collected for TCR β-sequencing are indicated by the gray arrow. Details of T cell expansion, cell sorting, and sequencing are described in Materials and Methods. C Negative control well, cultivated without Spike protein; +S - T cell expansions with added Spike protein. **(B)** IFNγ T cell response to Spike protein, measured by ELISPOT in vaccinated participants (n = 50) and PL (n = 19). **(C, D)** Intracellular production of IFNγ by CD4^+^ **(D)** and CD8^+^ **(C)** T cells after stimulation with Spike-derived peptide pools, measured by flow cytometry in vaccinated participants (n = 50) and placebo (PL, n = 19). **(E)** Spearman сorrelation between levels of IgG and T cell response, measured by flow cytometry and ELISPOT on the 14th day for all vaccinated participants. *p ≤ 0.05; **p ≤ 0.01; ***p ≤ 0.001; ****p ≤ 0.0001. In B-D Mann-Whitney U-test was used for testing statistical significance. The median is plotted on the graphs from independent experiments.

In this study, we investigated the clonal structure and dynamics of an antigen-specific T cell receptor repertoire in volunteers vaccinated with a single dose of the adenovirus vaccine Ad5-nCoV. We used blood samples from a cohort of 69 participants enrolled in a randomized, double-blind, placebo-controlled, single-dose phase 3 clinical trial of a recombinant adenovirus type 5 COVID-19 vaccine (NCT04540419). Peripheral blood was collected four times (days 0, 14, 28, and 6 months after vaccination), followed by PBMC isolation and downstream T cell assays. In our cohort, 50 donors received an Ad5-nCoV vaccine, and 19 were in a placebo group (PL) (**Figure 1A**).

Results from the clinical trial reported that a measurable cellular response, as determined by the ELISPOT assay at each time point, was evident as early as days 14 and 28 after vaccination (**Figure 1B**) (45). This demonstrated a significantly stronger T cell IFNγ response in the vaccinated compared to a placebo group (PL) that did not progress beyond the baseline of the assay. More specifically, the median number of spots after stimulation with the full-length recombinant Spike protein (**Figure 1B**) or the Spike-derived peptide pool (**Figure S1A**) was 107.6 and 110.8, respectively, while it was significantly (p-value < 0.0001) lower in the PL at day 14, with a median of 0 spots in both stimulation variants. At day 28, the median spots number for both measurements was 1.6 in the PL and 19.9 in the vaccinated group defined by ELISPOT assay (**Figures 1B**, **S1A**). The intensity of the T cell immune response was also measured by flow cytometric analysis of intracellular IFNγ and TNF production after stimulation with a Spike-derived peptide pool (**Figure S2A**). On days 14 and 28, IFNγ production by CD4^+^ and CD8^+^ T cells was significantly higher in the vaccinated group compare to the PL (p-value < 0.0001) (**Figures 1C, D**). This was also demonstrated for intracellular TNF production only for CD4^+^ on day 14, but not for CD8^+^ cells at any time-point (p-value = 0.0093 and 0.112, respectively) (**Figures S1B, S1C**).

When analyzing the dynamics of the T cell response, we observed a statistically significant increase in the response rate between Day 0 and Day 14 for all vaccinated individuals when assessed by ELISPOT or flow cytometry (p-value < 0.0001) (**Figures 1B-D**). We also observed a decrease in response rate by 6 months post-vaccination, but for most measurements the difference from Day 0 is maintained (p-value < 0.0001), for ELISPOT measurement with stimulation by peptide pools (p-value = 0.0005), but except for the assessment of TNF produced by CD4^+^ T cells (p-value = 0.9149) (**Figures S1A-C**).

We also found a correlation between the intensity of IFNγ secretion by T cells after Spike-derived peptide pool stimulation measured by ELISPOT and by flow cytometry on the 14th day (percentage of IFNγ^+^/CD4^+^ and IFNγ^+^/CD8^+^) (Spearman coefficients = 0.39 and 0.57, respectively, p-values = 0,0067 and 0,00003). Interestingly, we did not observe any correlation between cytokine production as measured by ELISPOT or flow cytometry and anti-RBD or anti-Spike antibody levels, taken from a previously published article on the results of a vaccine efficacy in clinical trial conducted in the same cohort of vaccinated individuals (**Figure 1E**) (45). We observed a lack of correlation between the T cell cytokine response assessed by flow cytometry and ELISPOT on day 14 and the antibody response (**Figure 1E**). This may be due to the fact that the peak of the T cell immune response occurs 14 days after vaccination, whereas the peak of the antibody response in our cohort occurred 28 days after vaccination (45).

### 2.2 Selection of participants with a strong T cell response

To characterize of the antigen-specific TCR repertoire after immunization, we identified T cell receptor clonotypes with a strong response to Spike protein. For this purpose, antigen-specific T cell expansion with recombinant full-length Spike protein was performed in replicates for each participant from blood samples collected on day 14. Unstimulated culture was used as a negative control (C-). After cultivation, cells were restimulated, and IFNγ-secreting CD4^+^/CD8^+^ cells were separated by fluorescence-activated cell sorting (FACS), followed by TCRβ sequencing using the Illumina platform (**Figure 1A**, red workstream). TCR repertoire of total PBMC fraction from all time points were used to evaluate the dynamics of identified T cell clonotypes (**Figure 1A**, green workstream). Samples obtained at 6th month after vaccination were subjected to Spike-specific T cell expansion to assess longevity of immune response (**Figure 1A**, yellow workstream).

Prior to unblinding the vaccine and placebo cohorts, we selected a group of target donors (TDs) for whom the first three time points were available and who had a detectable CD4^+^ and CD8^+^ T cell response after Spike-specific expansion (greater than 0.3% in the CD4^+^ or CD8^+^ population). An additional criterion that the number of sorted antigen-specific T cells exceeded 100 sorted IFNγ^+^ CD4^+^ and CD8^+^ T cells, was met by only 17 individuals due to the limited blood sample volume.

After unblinding, we confirmed that all individuals from the TD group were vaccinated, and the medians of responses of the TD group and of the remaining vaccinated group (RV, n = 33) were not statistically different (p-value > 0.05) in most of the assays (**Figures S1D-G**). As well as the entire cohort, TD demonstrated a significantly higher response to full-length Spike (**Figure S1D**) and Spike-derived peptide pools (**Figure S1E**) compared to PL. In the TD group, we also found a correlation between the intensity of IFNγ secretion after peptide pool stimulation measured by ELISPOT and intracellular IFNγ production of CD8^+^ cells by flow cytometry (Spearman coefficient = 0.74, p-value = 0.001) (**Figure S1H**). Similar to the correlations over all vaccinated participants (**Figure 1E**), for the 17 vaccinated individuals we detect a rather weak correlation for antibody and T cell responses, none of the correlation coefficients reach the p ≤ 0.001 or more significant (**Figure S1H**). This is consistent with literature data on the lack of correlation between T cell and humoral responses after vaccination with an adenoviral vector (46).

### 2.3 Clonality of Spike-specific T cell responses was higher in CD4^+^ than in CD8^+^ T cells after vaccination

Restimulation with Spike protein resulted in a dominance of CD4^+^ T cell response over CD8^+^ response, which corresponded to the difference in the number of sorted and sequenced cells (**Figures S2B-S2D**). The vaccinated group had a median of 0.46% IFNγ^+^ CD4^+^ cells, whereas the response in the PL group was 20-fold lower (median 0.002%, p-value < 0.0001) (**Figure S2C**). Similarly, the median CD8^+^ IFNγ^+^ in the vaccinated group was 0.18%, while the median in the PL group was 0.001% (**Figure S2C**).

**Figure 2:**
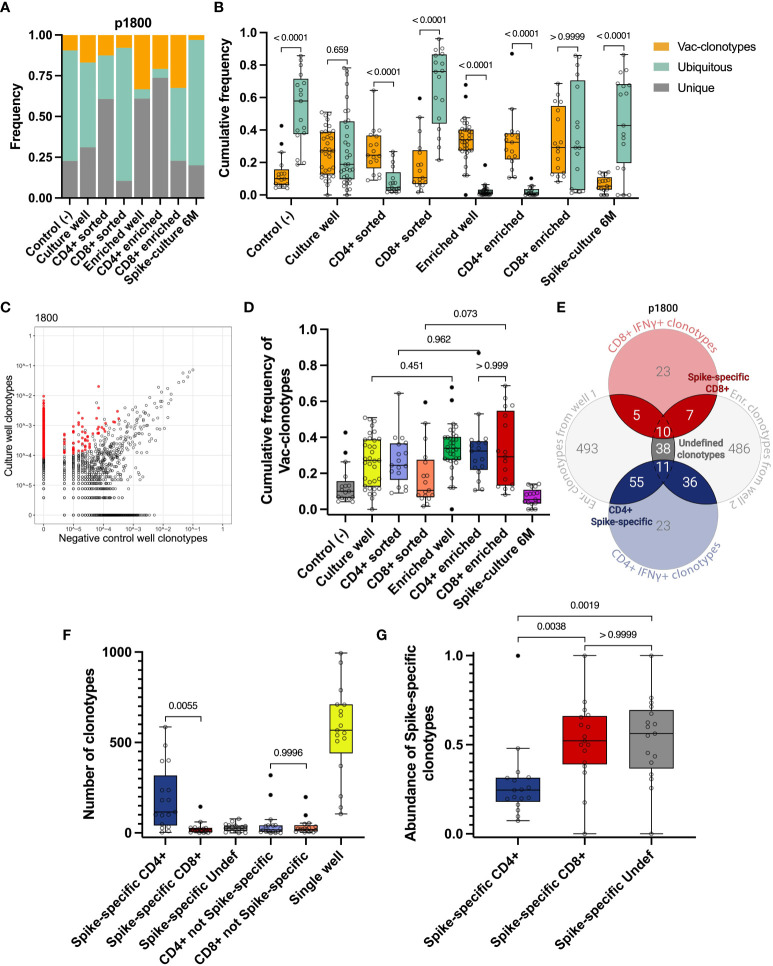
Identification of vaccine-induced Spike-specific clonotypes. **A, B** Cumulative frequency of Vac-clonotypes (orange), Ubiquitous (turquoise), or Unique (grey) in different samples of a representative donor (p1800) **(A)** or in all vaccinated donors (**B**, only Vac and Ubiquitous clonotypes are shown). Spike-expansion 6M - expanded Spike-specific culture from samples collected 6 months post-vaccination. **(C)** A representative enrichment plot for donor p1800, showing frequencies of CDR3β sequences in the culture well, stimulated by Spike protein versus untreated control culture. Red dots represent clonotypes that are enriched in the culture well. **(D)** The comparison of cumulative frequencies of individuals Vac-clonotypes in different fractions. **(E)** Venn diagram for representative donor illustrating the overlapping clonotypes in enriched fractions of the T cell after the Spike-specific expansion on day 14, with sequenced IFNγ^+^ T cell clonotypes. **(F)** Number of clonotypes in different groups as illustrated on E across donors. Undef - Undefined clonotypes. **(G)** Abundance of the Spike-specific clonotypes found in the total repertoire on day 14 for all donors. In **(B)** Mann-Whitney U-test was used for testing statistical significance. In **D**, **F**, **G**, one-way analysis of variance (ANOVA) followed by Tukey’s multiple comparison test was used. The median is shown on the graphs of independent experiments.

As the first step, we directly compared total CDR3 repertoires from all time points after vaccination to identify vaccine-specific clonotypes that were found only after vaccination but not at day 0 (*Vac-clonotypes*). Clonotypes found at all time points (including day 0) were considered as *Ubiquitous clonotypes*, and clonotypes found at only one time point were considered as *Unique clonotypes* (**Figure 2**). We estimated the relative frequencies of these clonotypes in expanded cultures and sorted fractions. For each donor, we evaluated the clonotypes as they appeared in the peripheral blood TCR repertoire of the vaccinated individuals. For each of the sequenced samples, we estimated the proportion of frequency of the proportions of one of the three groups of clonotypes.

This approach allowed us to detect a significant proportion of non-specific clonotypes in the samples. In particular, we observed that Ubiquitous and Unique clonotypes together occupied the majority of both Spike-specific expansions (expansions from Day 14 and 6 months) and sorted CD4^+^ and CD8^+^ IFNγ^+^ T cell populations in most donors (**Figures 2A**, **S3A-P**). At the same time, Vac-clonotypes typically represented less than half of the repertoire of expanded cultures. Wells with Spike-specific expanded cells contained predominantly Vac-clonotypes (**Figure 2B**). The same pattern was observed in sorted fractions of CD4^+^ but not CD8^+^ cells. Sorting of CD4^+^ IFNγ^+^ cells was effective in increasing the proportion of Vac-clonotypes, while sorted CD8^+^ cells still contained a significant share of Ubiquitous non-specific clonotypes. Ubiquitous clonotypes also dominated the negative control expansions, representing a median of 57.8% (from 18.8% to 85.7%) of the TCR repertoire (**Figure 2B**). We did not evaluate Ad5-specific responses in this study. Therefore, we understand that Ad5-specific T cell clonotypes could potentially be found among the Vac-clonotypes.

To perform an enrichment of vaccine-specific CD4^+^/CD8^+^ clonotypes, only TCRβ sequences associated with ≥4 UMI were considered. This means that the TCRβ sequence was derived from at least 4 distinct RNA molecules (see Materials and Methods). We also chose a 3-fold enrichment in the T cell expansion sample compared to the negative control expansion as a threshold (**Figures 2C**, **S2I**, **S2J**). These criteria significantly reduced the proportion of Ubiquitous clonotypes without substantial loss of Vac-clonotypes in all enriched fractions (**Figures 2A, B**). The trend of an increasing proportion of Vac-clonotypes after enrichment was also observed for CD4^+^ and CD8^+^ clonotypes in sorted fractions, but did not reach statistical significance (**Figures 2D**, **S2E**). The median of the cumulative frequency of Vac-clonotypes was approximately the same in CD4^+^ and CD8^+^ enriched subpopulations despite the greater variation in CD8^+^ frequencies for donors (**Figures 2D**, **S3**).

Thus, we have shown that the method of direct detection of antigen-specific clonotypes in peripheral TCR repertoires after vaccination introduces a significant proportion of clonotypes with uncertain specificity. Therefore, in order to more accurately determine the truly Spike-specific clonotypes that have arisen after vaccination, we have strengthened the criteria by which we select clonotypes. For each donor, clonotypes enriched in either of the two individual wells of Spike-specific expansions were overlapped with clonotypes found in the FACS-sorted fractions after restimulation. Clonotypes found in at least two of these fractions were further designated as Spike-specific. Depending on their presence in the CD4^+^ IFNγ^+^ or CD8^+^ IFNγ^+^ cells, they were further assigned to CD4^+^, CD8^+^, or Undefined T cell subpopulations (**Figure 2E**). Additionally, we found a median of 186 (from 36 to 629) total T cell clonotypes with a median of cumulative frequency in expanded cultures of 0.27 (from 0.067 to 0.76). Most clonotypes (from 3 to 586, median 116) were CD4^+^, the number of CD8^+^ clonotypes was significantly lower (from 1 to 145, median 17). We identified a median of 26 (ranging from 0 to 78) clonotypes that could not be assigned to either CD4^+^ or CD8^+^ subpopulations (Undefined). No significant difference was observed between the number of non-Spike-specific CD8^+^ and non-Spike-specific CD4^+^ clonotypes (**Figure 2F**).

Interestingly, despite the predominance of the number of Spike-specific CD4^+^ clonotypes, CD8^+^ clonotypes were more abundant, occupying more than 52% of the Spike-specific repertoire compared to 24% for CD4^+^ (**Figure 2G**). When analyzing the frequencies of Spike-specific clonotypes in the total repertoire of vaccinated donors on day 14, we found a difference between CD4^+^ and CD8^+^ for the mean frequency but not for the total frequency (p-value = 0.0708 and > 0.9999, respectively) (**Figures S2F, G**). Accordingly, CD4^+^ Spike-specific clonotypes show more diversity than CD8^+^, as estimated by the Shannon index (median 6.41 for CD4, median 3.43 for CD8) (**Figure S2G**).

### 2.4 The peak of immune response is reached on the 14th day after vaccination

**Figure 3:**
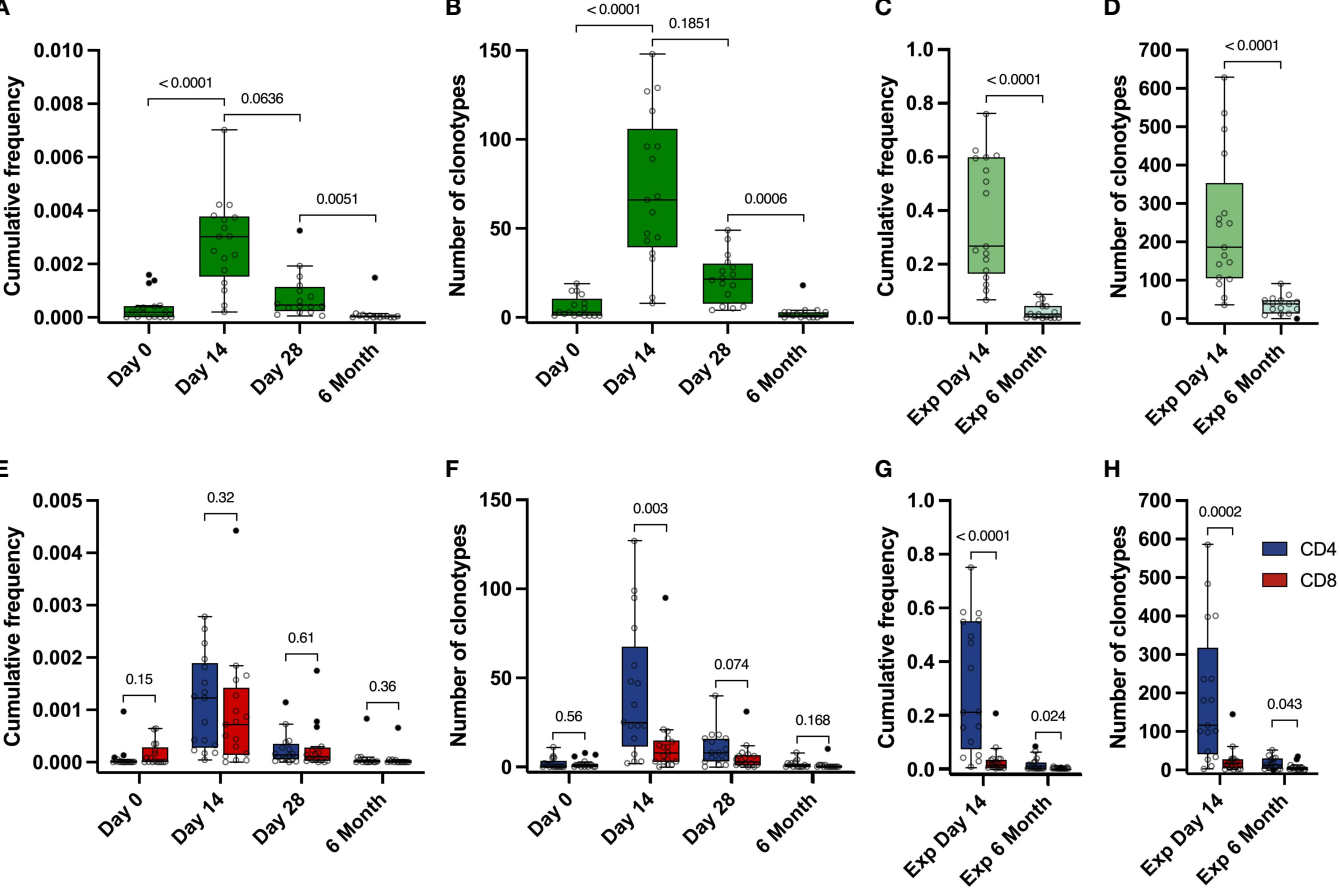
Dynamics of Spike-specific T cell response. **(A, E)** Dynamics of the cumulative frequency of **(A)** all Spike-specific clonotypes or **(E)** clonotypes identified as CD4^+^ (blue) and CD8^+^ (red) in the total repertoire at 4 time points. The values of the cumulative frequencies of clonotypes detected at each time point for each of the vaccinated individuals in the total peripheral blood repertoire are shown. Each point reflects the cumulative frequency of all clonotypes for one donor. **(B, F)** Dynamics of the number of all Spike-specific clonotypes **(B)** or CD4^+^ (blue) and CD8^+^ (red) clonotypes **(F)** found in the total repertoire at 4 time points. The values of the number of Spike-specific clonotypes detected in the peripheral blood of each vaccinated individual during sequencing are shown. Each point reflects the total number of clonotypes detected for one donor. **(C, G)** Cumulative frequency of all Spike-specific clonotypes **(C)** or CD4^+^ (blue) and CD8^+^ (red) clonotypes **(G)** detected in Spike-specific expansion at day 14 and in Spike-specific expansion at 6 months. D and H Number of all Spike-specific clonotypes **(D)** or CD4^+^ (blue) and CD8^+^ (red) clonotypes **(H)** detected in Spike-specific expansion at day 14 and in Spike-specific expansion at 6 months. For A and B, significant Kruskal-Wallis test followed by Dunn’s multiple comparison *post hoc* test was used. For C-H, Mann-Whitney U-test was used to test statistical significance.

To examine the dynamics of the Spike-specific T cell response after vaccination, we evaluated the number of Spike-specific clonotypes and their cumulative frequency in the total TCRβ repertoire for 17 donors in the TD cohort at each time point. The frequency of Spike-specific clonotypes peaked on day 14 after the vaccination (from 2,06×10^-4^ to 7,02×10^-3^, median 3,02×10^-3^), and decreased by 6 month (median 3,75×10^-5^) (**Figure 3A**). A similar dynamic was observed for the number of Spike-specific clonotypes: the median peaked at 66 (from 8 to 148) on day 14 and dropped to a median of 1 at month 6 (**Figure 3B**). The same overall T cell response pattern, peaking at 14 days and declining by month 6, was observed when assessing individual clonotype frequencies (**Figure S4C**), although for some of the clonotypes we observed a peak response at day 28.

Identified Spike-specific clonotypes from expanded cultures from day 14 were no longer detectable in the total repertoire by month 6. However, they still persisted in the blood and were detected after Spike-specific expansions. We were able to detect a substantial part of the Spike-specific clonotypes (from 9 to 91, median 38.5), with their median cumulative frequency in the expansion culture 1.46×10^-2^ (from 5.46×10^-4^ to 8.78×10^-2^), which is significantly lower than in the expanded T cell culture from day 14 (from 0.067 to 0.76, median of 0.27) (**Figures 3C, D**). In total, this represents approximately 13% of the total number of clonotypes (536 out of 4151) that were initially identified in the Spike-stimulated T cell cultures including CD4^+^, CD8^+^ as well as Undefined.

The cumulative frequencies of CD4^+^ and CD8^+^ Spike-specific clonotypes decreased, but the CD4^+^/CD8^+^ ratio remained stable during the post-vaccination observation period. (**Figure 3E**). At the same time, we observed a predominance of CD4^+^ clonotypes in the expansions, and the number of CD4^+^ clonotypes was higher than that of CD8^+^ clonotypes at each time point in both the total repertoire and in the Spike-specific expanded cultures. (**Figures 3F-H**). The mean frequency of CD4^+^ clonotypes was also higher than CD8^+^ in expanded T cell cultures, but not in the total repertoire (**Figures S4B, S4C**). Similarly, the total number and frequency of clonotypes for each donor peaked at day 14 in the CD4 and CD8 or Undefined subgroups (**Figures S4D-M**). In summary, Spike-specific CD4 showed higher TCR diversity but comparable frequencies in peripheral blood.

### 2.5 Identification of clonotypes specific to immunodominant epitopes

**Figure 4:**
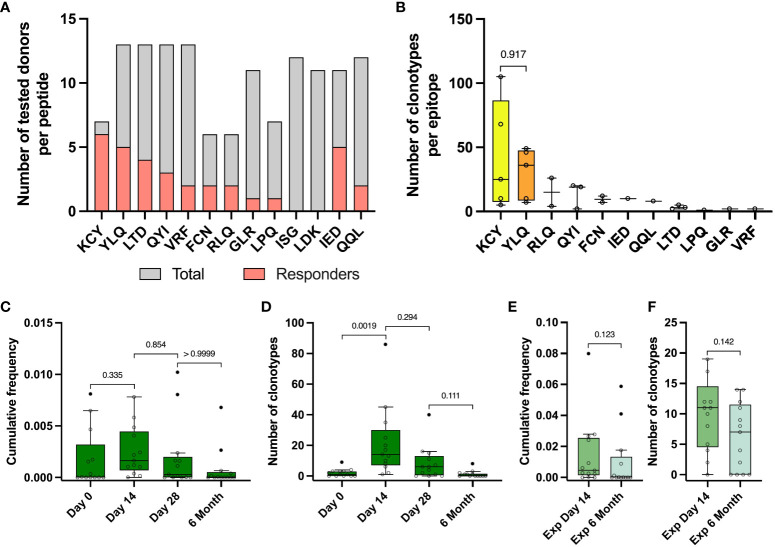
Identified epitope-specific clonotypes had the same dynamics as Spike-specific clonotypes. **(A)** For each epitope number of IFNγ- responders (pink) within the total number of screened donors (grey) is depicted. IFNγ production was measured with Elisa after restimulation of expansion derived from PBMC collected on day 14 with peptides. **(B)** Number of identified epitope-specific unique TCR clonotypes per donor per each epitope. **(C)** Dynamic of cumulative frequency of all epitope-specific clonotypes, found in the total repertoires in 4 time points. **(D)** Dynamics of a number of all epitope-specific clonotypes found in the total repertoires in 4 time points. **(E)** Cumulative frequency of all epitope-specific clonotypes, found in Spike-specific expansion on the day 14 and 6 months after vaccination. **(F)** The number of all epitope-specific clonotypes found in Spike-specific expansion on the 14th day and 6 months. The interquartile range is plotted on the graph. Mann-Whitney U-test (in **B**, **E**, **F**) and significant Kruskal-Wallis test followed by Dunn’s multiple comparison *post hoc* test (in **C** and **D**) were used to test statistical significance.

To evaluate epitopes of the Spike protein that induced an immune response in vaccinated participants, we compiled a set of peptides from the Spike protein, that were identified as immunogenic in previous studies of SARS-CoV-2 convalescents (21, 47). We selected 9 MHC class I peptides and 4 MHC class II peptides that were relevant for 13 of 17 donors from the TD group based on their HLA typing (**Table S2**, **Table S3**). Cells from each donor were stimulated with 4-13 peptides; peptide cocktail was selected based on individual combination of the donor’s HLAs and accounted for predicted HLA binding. Using PBMC collected on day 14, we performed an expansion with a peptide mix (**Figure 1**, blue area). After expansion, part of the cultures was stimulated with single peptides with an IFNγ ELISA (enzyme-linked immunosorbent assay) readout (**Figure 4A**). KCYGVSPTK (KCY) peptide was the most immunogenic – 6 out of 7 tested donors were positive (86%). YLQPRTFLL (YLQ) peptide was predicted as a binder for 13 donors, but YLQ-specific response was detected in only 5 who were carriers of the HLA-A*02:01 allele. Two peptides, LDKYFKNHTSPDVDL (LDK) and ISGINASVVNIQKEI (ISG), did not induce cell activation in any donor, so we excluded them from further analysis. In total, we assessed 11 peptides - 9 HLA class I peptides and 2 class II peptides, 1-3 peptides per donor (**Figure S5A**).

We stimulated the cultures with peptides that were immunogenic according to ELISA and performed an AIM (Activation-Induced Markers) assay (measurement of CD137 and CD69 expression in CD8^+^ cells and CD137 and CD134 in CD4^+^ T cells) with subsequent sorting of the activated fraction and TCRβ sequencing (**Figure S5B**). We also sequenced TCR from the total fraction of cultured cells after epitope-specific expansion (**Figure 1A**, grey arrow) to ensure correct assignment of clonotypes to the particular epitope by statistical enrichment analysis. The use of MHC tetramers would have significantly increased the sensitivity of the technique and allowed us to sort peptide-specific T cells with a higher degree of confidence. However, we hypothesized that we could simultaneously observe peptide binding to the different HLA alleles represented in our selected donors. Therefore, we decided that a better strategy would be to sort the cells after activation with peptides.

From 13 vaccinated donors, we identified a total number of 484 clonotypes specific to 11 epitopes, with a median of 30 clonotypes per donor (from 2 to 141). KCY and YLQ-specific T cell response demonstrated higher diversity in comparison to the other peptides, with a median of 25 and 36 identified clonotypes, respectively (**Figure 4B**) Surprisingly, the identified CD8^+^ Spike-specific clonotypes did not show a strong overlap with epitope-specific clonotypes recognizing peptides presented in MHC class I (15 out of 484 identified clonotypes) (**Figure S5C**). No overlap was found for CD4^+^ clonotypes. This may be explained by a higher dose of antigen or more efficient presentation of exogenous peptides added to cultures, resulting in non-specific stimulation of low affinity T cell receptors, regardless of the immunodominant properties of the peptides. In addition, simultaneous presentation of multiple immunodominant peptides from the Spike protein can alter the ratio of antigens presented.

Epitope-specific clonotypes showed the same dynamics of the number of detected clonotypes and their cumulative frequency at different time points as it was shown for Spike-specific clonotypes (**Figures 4C, D**). We detected from 1 to 86 (median 14) epitope-specific clonotypes on the day 14 after vaccination, with a median cumulative frequency of 1,64×10^-3^, comparable with the cumulative frequency of Spike-specific clonotypes on day 14 of 3,02×10^-3^ and up to 0-8 clonotypes (median 0 clonotypes) by 6 months (**Figure 4D**). Approximately one third of all epitope-specific clonotypes (172) were found in wells of Spike-specific expansion from PBMC collected on day 14. At least half of them (81 clonotypes) with a mean cumulative frequency of 0.009 were detectable in Spike-specific expansions at 6 months. However, we did not observe a statistical difference in the frequencies or numbers of epitope-specific clonotypes detected in expansions (**Figures 4E, F**).

### 2.6 Evaluation of publicity and homology of the identified Spike-specific clonotypes

To analyze the similarity of identified antigen-specific TCR clonotypes, we clustered Spike-specific TCR clonotypes (CD4^+^, CD8^+^, and Undefined) based on TCR β-chain CDR3 amino acid sequences. Clonotypes with no amino acid substitutions (Hamming distance = 0) found in different donors were marked as public; clonotypes with 1 or 2 substitutions (Hamming distance = 1 or 2) were marked as similar.

**Figure 5:**
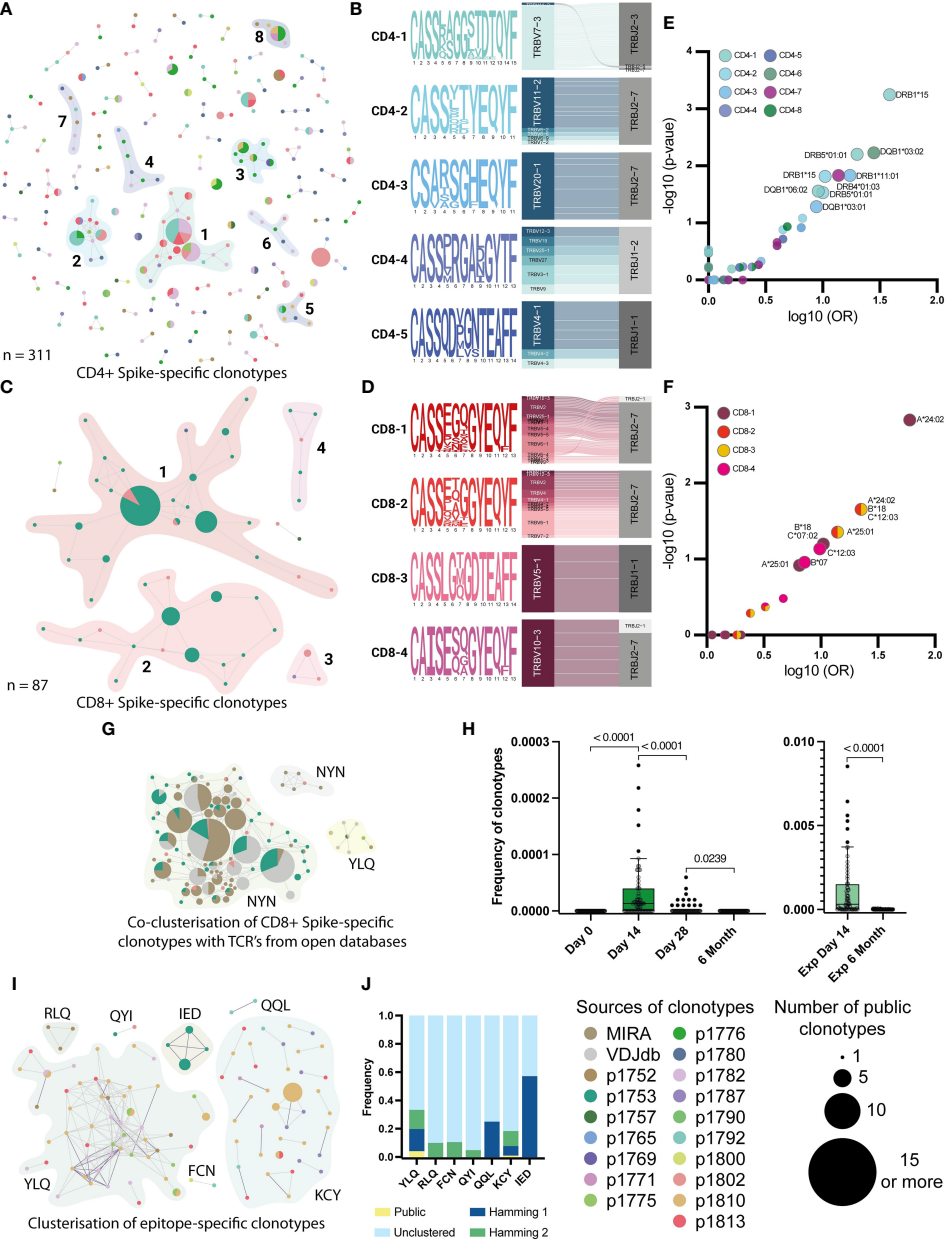
Clusterization by similarity of specific clonotypes. A and C Clusterisation of 311 CD4^+^ **(A)** and 87 CD8^+^ **(C)** Spike-specific clonotypes with Hamming distance = 1. Unclustered clonotypes are not shown. The size of nodes reflects a number of identical (public) sequences, colors indicate donors. Clusters of interest are named and highlighted. **(B)** and **(D)** position-weight matrices for CDR3β and V-J-genes usage (Sankey plots). Cluster numbers corresponds to numbers shown in **(A)** and **(C)**; **(E)** and **(F)** Volcano plot with the fold of the particular HLA in each cluster. Clusters are indicated by colors. Axes denote the decimal logarithm of the odds ratio of clusterization versus the negative decimal logarithm of the p-value (Fisher’s exact test). **(G)** Clusterization of CD8^+^ Spike-specific clonotypes with annotated sequences from databases. The colors indicate epitope specificity. **(H)** Dynamic of frequency of each NYN-specific clone, found in the total repertoires in 4 time points. Significant Kruskal-Wallis test followed by Dunn’s multiple comparison *post hoc* test (left graph) and Mann-Whitney U-test (right graph) were used to test statistical significance. **(I)** Clusters of CDR3-regions of all epitope-specific clonotypes with Hamming distance =2. Unclustered clonotypes are not shown. The size of nodes reflects a number of identical (public) sequences and color indicates donors. Clusters of interest are named and highlighted. **(J)** Normalized frequency of clonotypes with public (Hamming distance = 0), similar (Hamming = 1 and 2), or not similar (unclustered) CDR3 in the total repertoire from the day 14.

For the CD4^+^ clonotypes, we found 8 homology clusters, 5 of which contained public clonotypes (**Figure 5A**). For each of these 8 clusters, we also evaluated the V- and J-gene usage among the involved clonotypes and generated a CDR3 sequence logo. Most clusters (5 out of 8), such as CD4-1, -2, -3, -5, and -8, show dominant usage of a particular V-gene in combination with a particular J-gene (**Figures 5B**, **S6A**). Among 4 CD8^+^ clusters, 3 (CD8-1, -2, and -4) showed high publicity and similarity between 3 vaccinated donors (p1753, p1780, and p1802), with predominant usage of TRBJ2-7 (**Figures 5C, D**). We found no difference between the frequencies of CD8^+^ clustered and unclustered clonotypes in the total repertoires and in the Spike-stimulated cultures from samples collected on day 14, while the frequency of unclustered CD4^+^ clonotypes in the total repertoires was slightly higher than that of clustered clonotypes (p-value = 0.025) (**Figures S6B-E**).

We also examined the HLA restriction of clonotypes forming homology clusters (**Figures 5E, F**). In all patients with clustered CD4^+^ clonotypes, HLA-DRB1*15 was more prevalent than other alleles. In particular, the HLA-DRB1*15 allele was found in all donors whose clonotypes were in the CD4-1 cluster (p-value 0.0006, Fisher’s exact test). This allele was also present in 4 out of 6 donors contributing to the CD4-2 cluster (p-value = 0.015, Fisher’s exact test). For CD8^+^ clonotypes, HLA-A*24 was found in all 3 donors whose clonotypes belonged to clusters CD8-1, CD8-2 and CD8-3 (p-value = 0.0015, 0.02 and 0.02, respectively). For clusters CD8-2 and -3, there was also an association with HLA-B*18 and C*12 alleles in linkage disequilibrium with A*24 (p-value = 0.02). For the Undefined clonotypes, we found that HLA-C*07 and DRB1*07/1*15 were present in the majority of donors (**Figures S6F-H**). However, both are common alleles in the European population.

### 2.7 Expanding knowledge of epitope-specificity leveraging publicly available data

Previously, several studies have performed TCR sequencing of antigen-specific T cells from COVID-19 patients and mapped TCRs to their cognate epitopes. We collated data from the Multiplex Identification of T cell Receptor Antigen Specificity (MIRA) dataset (48) and the VDJdb database (http://vdjdb.cdr3.net) (49) with our Spike-specific clonotypes. Interestingly, for the CD8^+^ Spike-specific clonotypes, we found many similar CDR3 sequences in both databases, and similar clonotypes from the databases allowed us to merge 3 out of 4 clusters (CD8-1, -2, and -4) into a single cluster (**Figure 5G**). Most of the clustered clonotypes from the databases were annotated to be specific for the NYNYLYRLF (NYN) peptide from the Spike protein. This epitope is presented in HLA-A*24, which was found in all three donors whose clonotypes formed this cluster (**Table S2**). Most of the clustered NYN-specific receptors revealed the strong prevalence of V2, V6-1 and V10 genes. Therefore, we assume that all identified clonotypes that clustered with sequences from databases were also NYN-specific. We also evaluated the dynamics of the frequency of NYN-specific clonotypes in the total donor TCR repertoire across all time points and confirmed that these clonotypes emerge after day 14 and can be found in expansion with Spike protein after 6 months (**Figure 5H**).

When assessing the homology of the epitope-specific clonotypes, we observed strong similarity and publicity of YLQ-specific CDR3 sequences between donors and KCY-specific clustering. Other epitope-specific clonotypes revealed mutual similarity only when clustered with a Hamming distance = 2 (**Figures 5I, J**). Comparison of TCR sequences associated with specific epitopes in this study with annotated receptors from the databases revealed homologous receptors only for the YLQ epitope. Together, they formed three clusters containing clonotypes from 5 donors (**Figure S6I**). We also evaluated that all similar CDR3 clonotypes had two dominant V and two J genes. In particular, clonotypes from YLQ-2 use the V7-9 gene and many different J genes, with a preference for the J1-1 gene, whereas the other two clusters show the use of TRBJ2-2 paired with TRBV20-1 (YLQ-3) or with a set of different V genes (**Figure S6J**).

In summary, using external datasets, we were able to determine that YLQ-specific T cell receptors demonstrate extreme homology across subjects, while receptors specific for the other epitopes are much more diverse. We also found that many of the receptors in our dataset are most likely specific for the immunodominant NYN epitope, which was not originally included in our peptide panel.

## 3 Discussion

The aim of our study was to evaluate the T cell response induced by a vaccine based on the Ad5-nCoV adenoviral vector against SARS-CoV-2 carrying the Spike protein as immunogenic antigen. By analyzing the repertoire of T cell receptors in vaccinated volunteers, we assessed the diversity of the antigen-specific response and followed its dynamics from the time of vaccination until 6 months after vaccination. Our results demonstrate the robustness and diversity of T cell responses following vaccination and may be important for the future use and development of adenoviral vector-based vaccines.

Consistent with published data on the immune response to the Spike protein assessed after natural infection or vaccination, we confirmed that vaccination with the Ad5-nCoV vaccine induces a strong and durable T cell response (19, 20, 24). Previously published results demonstrating a humoral response to the vaccine show peak seroconversion at days 14 and 28 (45). We observed a similar pattern for the T cell response as assessed by ELISPOT and flow cytometry, but did not find a correlation between the T cell response and anti-RBD or anti-Spike antibody levels in our cohort. We also found that despite a decrease in the intensity of the T cell response after vaccination at 6 months, it remains higher than before vaccination. This is consistent with previously obtained data on the lack of correlation between CD8^+^ T cell response and antibody response to the Spike protein in adenovirus vaccinated subjects (46).

In this work, we identified CD4^+^ and CD8^+^ Spike-specific T cells generated after vaccination. We performed ex vivo antigen-specific expansions of peripheral blood T cells from samples collected 14 days after vaccination with the addition of full-length Spike protein and then sorted activated T cells after restimulation. Sequencing of TCR repertoires showed that there were significantly more Spike-specific CD4^+^ clonotypes than CD8^+^, but the average clonotype size for CD4^+^ was significantly smaller. Many studies have demonstrated high levels of CD4^+^ T cell activation following SARS-CoV-2 infection or vaccination. In particular, Mateus et al. described T cell responses in a cohort of mRNA-1273 (Moderna) vaccine recipients and suggested that the CD4^+^ T cell response predominated over CD8^+^ T cells after vaccination due to a pre-existing pool of cross-reactive CD4^+^ memory T cells (50). Other studies explained the prevalence of CD4^+^ responses with an initial low involvement of CD8^+^ cells or with the suboptimal experimental approach used in these studies (51–54). At the same time, sequencing of T cell receptors after vaccination with the Ad5-nCoV adenovirus vaccine in the study by Cao et al. showed that the specific CD8^+^ CTL clones undergo the greatest expansion after vaccination (55), which is consistent with our conclusion that the large clones of CD8^+^ cells may account for more than half of the donor Spike-specific repertoire.

Modern studies of the T cell repertoire of vaccinated or recovered COVID-19 patients allow qualitative assessment of the T cell response after immunization or determination of the antigen specificity of the T cell response. Most studies have focused on assessing the diversity of TCRs in COVID-19-recovered patients and comparing them with vaccinated, showing a gradual decline in T cell responses over time. The repertoire of SARS-CoV-2-specific TCRs in recovered patients is broader than in vaccinated individuals because vaccines carry a limited set of antigens, thereby limiting the repertoire of TCRs (56, 57), while vaccination promotes a broader repertoire specific for the Spike protein with more recognized antigens compared to recovered individuals (57). At the same time, it has been shown that vaccination can lead to activation and expansion of T cell clones that were not involved in the response to natural infection (58).

In this study, we also described that although the TCR repertoire decreases over time, it still maintains its clonality. We also described the differential dynamics in the number and frequency of Spike-specific CD4 and CD8 clonotypes. In addition, we showed that at least 13% of all unique Spike-specific clonotypes can be detected 6 months after vaccination following restimulation of peripheral blood T cells with full-length Spike protein. This is consistent with previously published work on the duration of T cell detection after vaccination (50, 51), but also extends the understanding of changes in the TCR repertoire of antigen-specific cells over time.

A potential limitation of the study is the small TD cohort and a potential bias introduced by the threshold set for the number of cells available for downstream analysis of Spike-specific T cell repertoires. However, our data did not indicate that the clonality and stability of the observed vaccine-specific responses were affected by the number of T cells sorted. This gives us confidence that the main finding of our study is valid for the entire study cohort.

An important component of this work was the homology analysis of T cell receptor clonotypes that recognize immunodominant epitopes of the Spike protein. Using CDR3 sequences annotated in databases, we identified with high confidence Spike-specific T cell receptors that potentially recognize the NYN epitope present in the HLA-A*24 allele (39). Interestingly, this epitope has been described to have different immunodominance in studies where it was identified either as a highly immunodominant epitope from the Spike protein or as a less immunogenic epitope present in the context of the HLA-A*24:02 allele (specifically compared to the QYIKWPWYI peptide) (39, 59, 60). We were also able to confirm that the YLQ epitope is recognized by receptors with a high degree of homology and a certain bias. At the same time, receptors recognizing other immunodominant epitopes from the current work do not show significant homology, which may be due to the diversity of recognition mechanisms of the HLA peptide complex or insufficient annotation in the databases, which can only be corrected by performing multiple antigen-specific T cell expansions with further TCR sequencing.

By tracking the diversity and evolution of T cell clonotypes over time, we can gain a better understanding of the long-term protective potential of vaccines. For the two immunodominant epitopes NYN and YLQ described above, we have observed long-lasting responses of up to 6 months. However, mutations in these epitopes reduce their recognition, for example a mutation leading to the replacement of proline by leucine at position 4 in YLQ (61). Such mutations may occur in the new strain: the substitution of leucine for arginine at position 4 in the NYNYLYRLF epitope is present in more than 95% of all SARS-CoV-2 Delta sequence variants in GISAID. However, it is unknown whether this epitope can be recognized by other TCR motifs and how much the mutation affects the immunodominance of this peptide (59). Accordingly, Spike-specific T cell responses elicited by the Ad5-nCoV vaccine targeting these immunodominant epitopes may be significantly reduced upon further interaction of the vaccine recipient with novel strains of SARS-CoV-2.

In addition to TCRs recognizing immunogenic epitopes, we identified many TCRs of unknown specificity that expanded and contracted with the epitope-specific T cell receptors. These receptors may recognize additional, even more immunodominant epitopes of the Spike protein or alternatively target vector-specific epitopes (Ad5 vector epitopes). Further studies are needed to unravel this, including comparisons of our data with TCR repertoires elicited by vaccines with the same adenoviral backbone and different transgene.

In conclusion, our study provides important insights into the durability and diversity of T cell responses induced by the Ad5-nCoV vaccine. It may be useful for current vaccine use and further development of novel adenoviral vector-based vaccines. By analyzing the response to the Spike protein as a model antigen, we demonstrated that the adenoviral vaccine induces a broad repertoire of T cell clonotypes that persist over time, consistent with the durability of T cell responses observed in natural infection and after vaccination with RNA-based vaccines.

## 4 Materials and methods

### 4.1 Human subjects

Sixty-nine healthy volunteers from Moscow, Russia, were recruited for a single-dose, randomized, double-blind, placebo-controlled, parallel-group, Phase 3 clinical trial (Prometheus) of the Ad5-nCOV vaccine (ClinicalTrials.gov: NCT04540419). A more detailed description of the participant cohort can be found in Lioznov et al. (45). 50 donors from the vaccinated group and 19 from the placebo group were enrolled in the T cell immune response study. For this cohort, blood samples were collected on day 0 (the day of vaccination) and 14, 28 days, and 6 months after vaccination.

Day 0 blood samples were collected in September and October 2020. According to Lioznov et al. (45), each participant underwent a thorough screening process within 1-10 days prior to receiving either the Ad5-nCoV vaccine or placebo on Day 0. Screening included real-time polymerase chain reaction (PCR) swab to detect SARS-CoV-2 RNA, and immunoglobulin M (IgM) and immunoglobulin G (IgG) antibody tests for SARS-CoV-2 to ensure negative results. A detailed medical history was obtained from each participant, including any history of COVID-19 symptoms and close contact with individuals suspected or confirmed to have SARS-CoV-2 infection. One vaccine recipient was detected as COVID-19 positive during the clinical study between 28 days and 6 months. His post-illness T cell response results were excluded from further analysis.

The vaccine efficacy study initially enrolled 496 volunteers at 6 sites across the country. Of these, 495 formed the full analysis set, which was defined as the main population for the vaccine efficacy analysis according to Lioznov et al. (45). For the evaluation of the humoral response, the ELISA assay was performed by the group of Lioznov et al. and the data were taken from the published article (45). For the analysis of the cellular immune response, a subset of 69 participants from the full analysis set was used for the immunogenicity analysis population (Ad5-nCoV group, n = 50 volunteers; Placebo group, n = 19). These were the participants who visited the Moscow clinic site where the cellular immune response analysis was performed.

### 4.2 Peripheral blood mononuclear cell isolation

7 mL of venous blood from healthy donors was collected into Li-heparin tubes (VACUETTE) and subjected to Ficoll (Paneco; Р050Е) density gradient centrifugation (400 x g, 30 min). Isolated PBMCs were washed with ice-cold PBS containing 2 mM EDTA (Serva; 3976102) and used for multiple assays or frozen in Fetal Bovine Serum containing 7% DMSO (Sigma; 472301). Venous blood was collected at 4 time points to assess antibody and T cell response parameters at predetermined time points as part of a clinical efficacy study of the Ad5-nCoV vaccine.

### 4.3 Flow cytometry

Freshly isolated PBMCs were plated in a 96-well plate (Sarstedt; 83.3925.500) at a density of 1.5 × 10^6^ cells/well in RPMI 1640 culture medium (Thermo; 31870025) supplemented with 1 mM sodium pyruvate (Thermo; 11360039), 5% normal human A/B serum, 1 mM L-glutamine (Thermo; 25030-024) and SARS-CoV-2 S protein-derived peptide pools (Miltenyi Biotec; 130-126-701) (final concentration 1 mM) followed by 1h incubation 37°C, 5% СО2. GolgiPlug (BD Biosciences; 555028) was added for 5h incubation (37°С, 5% СО2). Cells were stained for surface markers and viability using Cytofix/Cytoperm™ Fixation/Permeabilization Kit (BD Biosciences; 555028) followed by incubation with antibodies for 10h at 4°C.

Surface staining of PBMCs was performed for 20 minutes at 4°C with the following antibodies in 100 mcl PBS containing 2 mM EDTA and 0.5% BSA: CD3-AF700 (0.6 mcl; clone OKT3; Sony; 2186700), CD4-BV510 (2.5 mcl; clone OKT4; Sony; 2187220), CD8-PerCP/Cy5.5 (1.25 mcl; clone RPA-T8; Sony; 2105160). Viability staining was then performed with FVD780 eFluor 780 (eBioscience; 65-0865-14) according to the manufacturer’s standard protocol: cells were washed twice with protein-free PBS and resuspended in 100 mcl PBS with 0.1 mcl FVD780, followed by incubation at 4°C for 30 minutes. Cells were then washed with PBS containing EDTA and BSA and resuspended in Fixation/Permeabilization solution (BD Biosciences; 555028). Intracellular cytokine staining was performed for 10 hours at 4°C using the following antibodies in 100 mcl BD Perm/Wash™ buffer: IFN-γ-PE-Cy7 (0.1 mcl; clone B27; BD Biosciences; 560924), TNF-PE (0.3 mcl; clone Mab11; BD Biosciences; 559321). Cells were analyzed on a FACS Aria III cell sorter (BD Biosciences) with 3 lasers (405nm, 488nm, 633nm). FlowJo software (version 10.6.1) was used for analysis.

### 4.4 IFNγ ELISPOT

Measurement of antigen-specific IFNγ production by T cells was performed using the ImmunoSpot human IFNγ single-color ELISPOT kit (CTL) with a 96-well nitrocellulose plate precoated with human IFNγ capture antibody. Freshly isolated PBMCs were plated at a density of 3 × 10^5^ cells/well in duplicate in CTL test medium and pulsed separately with SARS-CoV-2 Spike protein and SARS-CoV-2 S protein-derived peptide pools (Miltenyi Biotec) at a final concentration of 10 μg/mL and 1 μM, respectively, in serum-free test medium (CTL) containing 1 mM GlutaMAX (GIBCO) at a final volume of 200 μL/well. Plates were incubated for 18 hours at 37°C in 5% CO2. Assays were then performed according to the manufacturer’s instructions. Briefly, plates were washed twice with PBS and then twice with PBS + 0.05% Tween-20, followed by incubation with a biotinylated anti-human IFNγ detection antibody for 2 hours at room temperature (RT). Wells were washed three times with PBS + 0.05% Tween-20 and streptavidin-AP was added for 30 minutes at RT. After several washes, the colorimetric reaction was initiated by adding substrate components for 15 min at RT. The reaction was stopped by gently rinsing the plate with tap water. Spots were counted on the CTL ImmunoSpot Analyzer using ImmunoSpot software. The negative control used for ELISPOT evaluations was the PBMC wells from the tested human subjects at the same concentration as the experimental wells, but without the added antigen or stimulant of the positive control (phorbol 12-myristate 13-acetate (PMA) and ionomicyn). The negative control was subtracted from each value as background.

### 4.5 T cell expansions

For rapid *in vitro* expansion with Spike protein, PBMCs were collected from 50 donors on day 14 after vaccination. Briefly, 3 × 10^6^ cells were plated at a density of 1 × 10^6^ cells/well in three separate wells of a 24-well suspension plate (Sarstedt; 83.3922.500) and incubated for 8-10 days in RPMI 1640 culture medium supplemented with 10% normal human A/B serum, 1 mM sodium pyruvate, IL-7 (25 ng/mL; 130-095-363), IL-15 (40 ng/mL; 130-095-765), and IL-2 (50 ng/mL; 130-097-743) (Miltenyi Biotec) at a final volume of 2 ml/well. Half of the medium was replaced on days 3, 5, and 7. A recombinant SARS-CoV-2 Spike protein (final concentration 20 μg/mL) was added to two wells on day 0, and the third well was used as a negative control. After expansion, we performed culture restimulation with autologous PBMCs frozen from Day 0 with the addition of Spike protein. This is described in more detail in section 4.7. For peptide-specific rapid *in vitro* expansions, we used PBMCs from 13 donors of the selected cohort, collected on day 14 after vaccination. Briefly, 3-5 × 10^6^ cells were plated at a density of 1-3 × 10^6^ cells/well in a 24-well suspension plate (Sarstedt; 83.3922.500) and incubated for 8-10 days in RPMI 1640 culture medium supplemented with 10% normal human A/B serum, 1 mM sodium pyruvate, IL-7 (25 ng/mL; 130-095-363), IL-15 (40 ng/mL; 130-095-765) and IL-2 (50 ng/mL; 130-097-743) (Miltenyi Biotec) at a final volume of 2 ml/well. Half of the medium was replaced on days 3, 5 and 7. A combination of SARS-CoV-2 Spike protein peptides in DMSO (Sigma-Aldrich; 472301) or MES buffer (Sigma-Aldrich; M3671) (final concentration of each peptide in the medium = 10 ng/mL) was added on day 0. After peptide-specific expansion, we restimulated part of the cultures by addition of peptides only and assessed the response by IFNγ ELISA (section 4.11). The remaining fraction was stimulated for AIM assay and further sorting (section 4.12). Six months after vaccination, PBMCs from 16 donors were collected and used for rapid *in vitro* expansion with Spike protein. 2-16 × 10^6^ cells were plated at a density of 1× 10^6^ cells/well in separate wells of a 24-well suspension plate (Sarstedt; 83.3922.500) and incubated for 8-10 days in RPMI 1640 culture medium supplemented with 10% normal human A/B serum, 1 mM sodium pyruvate, IL-7 (25 ng/mL; 130-095-363), IL-15 (40 ng/mL; 130-095-765), and IL-2 (50 ng/mL; 130-097-743) (Miltenyi Biotec) at a final volume of 2 ml/well. Half of the medium was replaced on days 3, 5, and 7. A recombinant SARS-CoV-2 Spike protein (final concentration 20 μg/mL) was added on day 0.

### 4.6 Expression and purification of recombinant proteins

Sequence encoding ΔFurin variant of SARS-CoV-2 S protein (amino acids 1–1213), a truncated variant that contains the ectodomain of S protein (i.e., recombinant S protein ectodomain) along with a C-terminal Gly-Gly-6xHis tag was subcloned into the pMCAG-2T vector using the GeneArt Type IIs Assembly Kit, BbsI (Thermo Fisher Scientific), according to the manufacturer’s instructions. Briefly, recombinant SARS-CoV-2 Spike protein-His6 was expressed in Expi293F cells (Thermo Fisher Scientific) maintained in BalanCDTM HEK293 medium (FujiFilm Irvine Scientific) supplemented with GlutaMax (Thermo Fisher Scientific) and 10 ug/ml gentamicin in ReadyToProcess WAVE™ 25 rocking bioreactor with disposable Cellbag 10L (Cytiva) (working volume of 5 liters). Cell culture was maintained at 37˚С (or 30˚С after transfection), flow rate of gas mix 0.5 l/min with 5% CO2, 25 RPM rocking speed. For transfection of 1 liter of cell culture 3 mg of linear PEI25K (Polysciences) and 1 mg of plasmid DNA dissolved in Opti-MEM™ I medium (Thermo Fisher Scientific) were used. Transfection was set up when viable cell density of 2.8*10^6^cells/ml was reached. Starting the next day after transfection and to the 4rd day BalanCD HEK293 Feed (FujiFilm Irvine Scientific) supplemented with 4 mM GlutaMAX was added. The second day after transfection sodium valproate (Sigma) was added to a final concentration of 4 mM. Cell growth was terminated 7 days after transfection. Cells were harvested at 4000 g, the conditioning medium was filtered through 0.45 µ PES filter, concentrated 20 times, and diafiltered against PBS pH 7.4 with 1M NaCl, 50 mM imidazole, 1mM DTT and 1mM PMSF (buffer A) using the ÄKTATM flux tangential flow filtration system (Cytiva, filter cartridge UFP-10-C-4X2MA). 25 volumes of concentrate were mixed with 1 volume of Ni-IMAC Sepharose FF (Cytiva) resin (QIAGEN) and agitated at 10°C overnight. The resin mixture was packed on a Vantage^®^ L 22 x 250 column (Millipore), washed buffer A and eluted with buffer A, containing 200 mM imidazole. The eluate was dialyzed 3 times against 200 volumes of PBS using Slide-A-Lyzer dialysis cassettes (20K MWCO, Thermo Fisher Scientific). The Spike protein used in this study was based on the genomic sequence of the first isolate, Wuhan-Hu-1, which was released on January 10, 2020 (GenBank: MN908947.3) (21).

### 4.7 IFNγ-secretion assay

Measurement of IFNγ secretion in CD4^+^ and CD8^+^ T cells was performed using the IFN-γ secretion assay-detection kit (Miltenyi Biotec; 130-090-762) according to the manufacturer’s protocol. Briefly, after expansion with recombinant SARS-CoV-2 Spike protein, cells were pooled and resuspended in RPMI 1640 culture medium (GIBCO) supplemented with 5% normal human A/B serum and 1 mM sodium pyruvate (GIBCO) and plated at a density of 1-10 × 10^6^ cells/ml. Cells were treated with 20 μg/mL recombinant SARS-CoV-2 Spike protein for 16 hours and with cells from the same donor frozen on day 0 at a ratio of 1:2-1:4 used as feeder, followed by incubation with IFNγ Catchmatrix reagent (Miltenyi Biotec; 130-090-762) for 5 minutes at 4°C. After the restimulation, 3 × 10^6^ cells of the restimulated expansion were then transferred to warm medium (37°C) for 45 minutes to reinitiate IFNγ secretion, washed with ice-cold PBS containing 2 mM EDTA and 0.5% BSA, and stained with surface markers in 100 mcl: CD3-AF700 (0.6 mcl; clone OKT3; Sony; 2186700); CD4-FITC (0.6 mcl; clone RPA-T4; Sony; 2102690); CD8-PerCP/Cy5.5 (1.25 mcl; clone RPA-T8; Sony; 2105160)) and IFNγ detection antibody-APC (10 mcl; Miltenyi Biotec; 130-090-762) for 10 minutes at 4°C. Viability staining was performed after staining of surface markers with FVD780 eFluor 780 (eBioscience; 65-0865-14) according to the manufacturer’s standard protocol as described above. CD4^+^IFNγ^+^ and CD8^+^IFNγ^+^ populations were sorted directly into TRIzol reagent (Thermo Fisher Scientific) using a FACS Aria III cell sorter (BD Biosciences) with 3 lasers (405nm, 488nm, 633nm). Data were analyzed using FlowJo software (version 10.6.1).

### 4.8 TCR repertoire sequencing

RNA from RLT-lysed cells was extracted using the RNeasy mini kit (Qiagen) according to the manufacturer’s protocol. cDNA libraries were generated using the Human RNA TCR Multiplex kit (MiLaboratories) according to the manufacturer’s protocol.

TCR libraries were prepared from T cell RNA as previously described (62). Briefly, RNA was isolated from Trizol reagent (Thermo Fisher Scientific) using Phasemaker Tubes (Thermo Fisher Scientific), the cDNA synthesis reaction for TCR β chains was performed with a primer to the C-terminal region and SMART-Mk, which provides a 5′ template-switch effect and contains a sample barcode for contamination control as well as a unique molecular identifier. TCR libraries were generated using the human multiplex TCR kit (MiLaboratories) according to the manufacturer’s instructions. Sequencing was performed on an Illumina MiSeq or NextSeq platform. TCR repertoire data were analyzed using MIXCR (63), MIGEC (64), and VDJtools software (65). Reads belonging to the same Molecular Identifier Group (MIG) were determined by tagging with the same Unique Molecular Identifiers (UMIs). The identical and highly similar sequences from collapsed MIGs were considered to represent unique clonotypes and were further included in the analysis. The output of the sequencing data for each sample is shown in **Table S1**.

### 4.9 HLA genotyping

For most donors, HLA genotyping was performed using the One Lambda ALLType kit (Thermo Fisher Scientific), which uses multiplex PCR to amplify the complete HLA-A/B/C gene sequences and from exon 2 to the 3′ UTR of the HLA-DRB1/3/4/5/DQB1 genes. Prepared libraries were run on an Illumina MiSeq sequencer using a standard flow-cell with 2 × 150 paired-end sequencing. Reads were analyzed using One Lambda HLA TypeStream Visual Software (TSV), version 2.0.0.27232, and the IPD-IMGT/HLA database 3.39.0.0. Other donors were HLA genotyped by Sanger sequencing for the HLA-A, B, C, DRB1, and DQB1 loci using Protrans S4 and S3 reagents. PCR products were prepared for sequencing using BigDye Terminator v1.1 (Thermo Fisher Scientific). Capillary electrophoresis was performed on a Nanophore 05 Genetic Analyzer.

### 4.10 SARS-CoV-2 Spike peptides

Putative 13 epitopes of Spike protein were included in the analysis if they were binders (rank < 2) according to NetMHCpan 4.1 (Reynisson, Alvarez, Paul, Peters, & Nielsen, 2020). Detailed information on selected peptides is provided in **Table S3**. The predicted proteasomal cleavage score of the C-terminal amino acid was estimated using NetChop 3.1 (66). Peptides (at least 95% purity) were synthesized either by Peptide 2.0 or by the Shemyakin-Ovchinnikov Institute of Bioorganic Chemistry RAS. Peptides containing Cys and/or Met were diluted in a PBS/isopropanol mixture (1:1 v/v) at concentrations up to 10-25 mM. Other peptides were diluted in DMSO (Sigma-Aldrich) up to 30-40 mM.

### 4.11 ELISA

Analysis of epitope-specific T cell responses was performed using an IFN-γ ELISA kit (Vector-Best). After expansion with a mixture of peptides, PBMC were combined and resuspended in AIM V culture medium (GIBCO) and plated at a density of 1 × 10^5^ cells/well in a 96-well U-bottom plate. Cells were treated for 16 hours with 1mcM of individual peptides restricted in the donor’s HLA. The conditional medium was used to perform assays according to the manufacturer’s instructions. Optical density was measured using a Multiskan™ FC Microplate Photometer (Thermo) on a 450 nm filter and a 650 nm filter.

### 4.12 Activation-induced markers assay

The presence of epitope-specific T cells was estimated using the Activation Induced Markers (AIM) assay. Briefly, after expansion with a mixture of peptides, PBMC were combined and resuspended in RPMI 1640 (GIBCO) supplemented with 5% normal human A/B serum and 1 mM sodium pyruvate (GIBCO) and plated at a density of 1 × 10^6^ cells/well in a 96-well U-bottom plate. Cells were treated for 24 hours with 1 mcM of individual peptides restricted in the donor’s HLA. Cells were then washed with PBS containing 2 mM EDTA and 0.5% BSA and stained for surface markers along with AIM markers in 100 mcl buffer for 20 minutes at 4°C: CD3-AF700 (0.6 mcl; clone OKT3; Sony; 2186700), CD4-BV510 (2.5 mcl; clone OKT4; Sony; 2187220), CD8-APC (1.25 mcl; clone SK1, BD; 345775), CD137-PE (1.25 mcl; clone 4B4-1; Sony; 2149020), CD69-FITC (2.5 mcl; clone FN50; Sony; 2154520) and OX40(CD134)-BV650 (2.5 mcl; clone ACT35; BD Biosciences; 563658). Viability staining was then performed using FVD780 eFluor 780 (eBioscience; 65-0865-14) according to the manufacturer’s standard protocol as described above. CD4^+^CD134^+^CD137^+^ and CD8^+^CD69^+^CD137^+^ populations were sorted directly into RLT reagent (Qiagen) using a FACS Aria III cell sorter (BD Biosciences) with 3 lasers (405nm, 488nm, 633nm). Data were analyzed using FlowJo software (version 10.6.1).

### 4.13 TCR repertoire analysis

Enriched CD8/CD4^+^ clonotypes were defined as a fraction with a frequency at least 3 times higher in CD4^+^/CD8^+^ INFg^+^ than in the negative control population (C-), with a size of at least 4 UMI, and with a frequency at least 20 times higher in the Spike-specific expansion than in the total at 6 months. Clonotypes enriched in the Spike-specific wells of the T cell expansion were defined as a fraction with a frequency significantly higher in the well than in the C- population (at least 5-fold higher and p-value < 10^-5^ (Fisher exact test without adjustment)) and with a frequency at least 20-fold higher in the Spike-specific expansion than in the total at 6 months.

Pooled AIM samples were demultiplexed by intersection with clonotypes from the total PBMC samples. Only the top 75% most frequent clonotypes from the total samples and the top 25% from the pooled samples were used. Antigen-specific clonotypes were defined as populations found in the whole sample from only one donor or populations with frequencies at least 10 times higher in the whole sample from one donor than from another. Clonotypes with multiple antigen specificities and clonotypes with a frequency in Spike-specific expansion at 6 months less than 20 times higher than the total at 6 months were removed.

Epitope-specific TCR sequences were matched to VDJdb and ImmunoCODE datasets using the VDJmatch tool, allowing maximum Hamming distance = 2. Graphs were plotted using the “igraph” R package version 1.2.6.

### 4.14 Quantification and statistical analysis

Shannon diversity indices were calculated using python3. All data comparisons and Spearman correlations were performed using GraphPad Prizm 9 software. The association between the presence of cluster-related TCRs and HLA alleles was calculated by the Fisher exact test using the SciPy python3 library. The donor-specific set of peptides predicted to bind to HLA class I (8-11 amino acids) and class II (15 amino acids) was calculated using NetMHCpan 4.1. Data are presented as median ± IQR; *p < 0.05, **p < 0.01, ***p < 0.001, ****p ≤ 0.0001. A p-value < 0.05 was considered statistically significant.

## Data availability statement

The datasets presented in this study can be found in online repositories. The names of the repository/repositories and accession number(s) can be found below: https://www.ebi.ac.uk/ena, PRJEB71810.

## Ethics statement

The studies involving humans were approved by National Research Center for Hematology ethical committee. The studies were conducted in accordance with the local legislation and institutional requirements. The participants provided their written informed consent to participate in this study.

## Author contributions

SS: Conceptualization, Formal analysis, Investigation, Methodology, Project administration, Visualization, Writing – original draft, Writing – review & editing. AK: Formal analysis, Investigation, Software, Visualization, Writing – review & editing. KZ: Formal analysis, Investigation, Methodology, Software, Writing – review & editing. IZ: Investigation, Validation, Writing – review & editing. AS: Investigation, Writing – review & editing. YS: Investigation, Writing – review & editing. NS: Investigation, Writing – review & editing. IP: Investigation, Writing – review & editing. AT: Investigation, Software, Validation, Writing – review & editing. DR: Investigation, Writing – review & editing. IS: Investigation, Writing – review & editing. DC: Funding acquisition, Investigation, Writing – review & editing. DK: Investigation, Writing – review & editing. OS: Investigation, Writing – review & editing. EK: Investigation, Writing – review & editing. VD: Resources, Writing – review & editing. AA: Resources, Writing – review & editing. AB: Funding acquisition, Writing – review & editing. GE: Conceptualization, Funding acquisition, Methodology, Project administration, Supervision, Validation, Writing – review & editing.
