# Supplementary material for: Clonal structure and the specificity of vaccine-induced T cell response to SARS-CoV-2 Spike protein
Source: Front Immunol. 2024 Apr 2;15:1369436. doi: 10.3389/fimmu.2024.1369436 (PMC11018901; doi:10.3389/fimmu.2024.1369436)
Supplement: Supplementary file 1 [file DataSheet_1.docx]

Supplementary Material

## Supplementary Figures


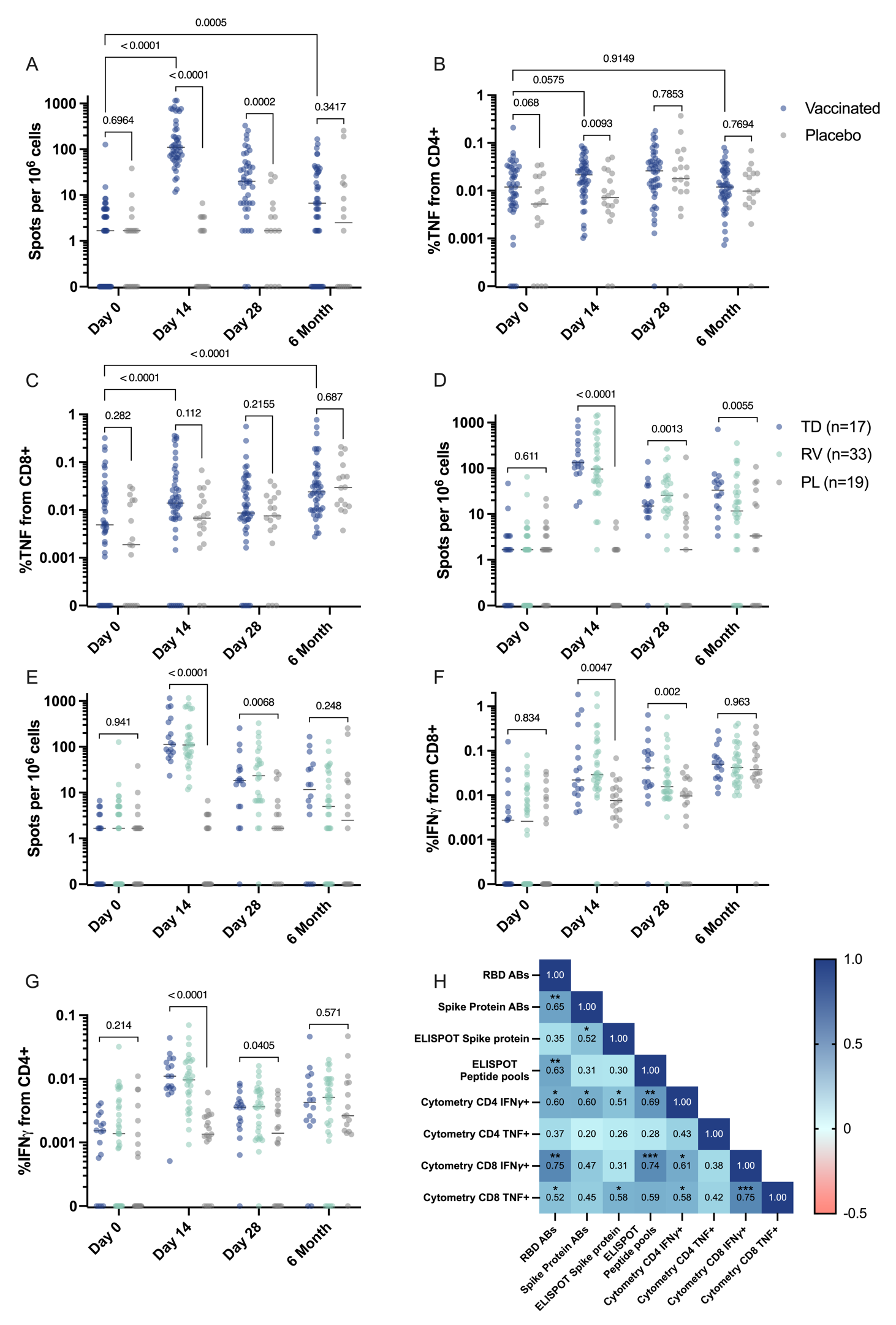


**Supplementary Figure 1. T cell response in vaccination and placebo groups after the unblinding of cohorts.**

**A** - T cell response to Spike-derived peptide pools, measured by IFNγ ELISPOT in vaccinated participants (n = 50) and PL (n = 19).

**B and C** - Intracellular production of TNF by CD4^+^(B) and CD8^+^(C) T cells after stimulation with Spike-derived peptide pools, measured by flow cytometry in vaccinated participants (n = 50) and PL (n = 19).

**D and E** - Response to Spike protein (D) and to Spike-derived peptide pools (E), measured by ELISPOT in PL and two groups of vaccinated participants: target donors (TD, n=17), whose samples we subsequently used for TCR β-sequencing and the remaining vaccinated (RV, n=33).

**F and G** - Intracellular production of IFNγ by CD4^+^(G) and CD8^+^(F) T cells after stimulation with peptide pools, measured by flow cytometry in PL, TD and RV.

**H** - Spearman сorrelation between levels of IgG and T cell response, measured by flow cytometry and ELISPOT on the 14th day in the TD group. *p ≤ 0.05; **p ≤ 0.01; ***p ≤ 0.001; ****p ≤ 0.0001.

In A-G the Mann-Whitney U-test was used to test statistical significance. The median is shown in the plots of independent experiments.


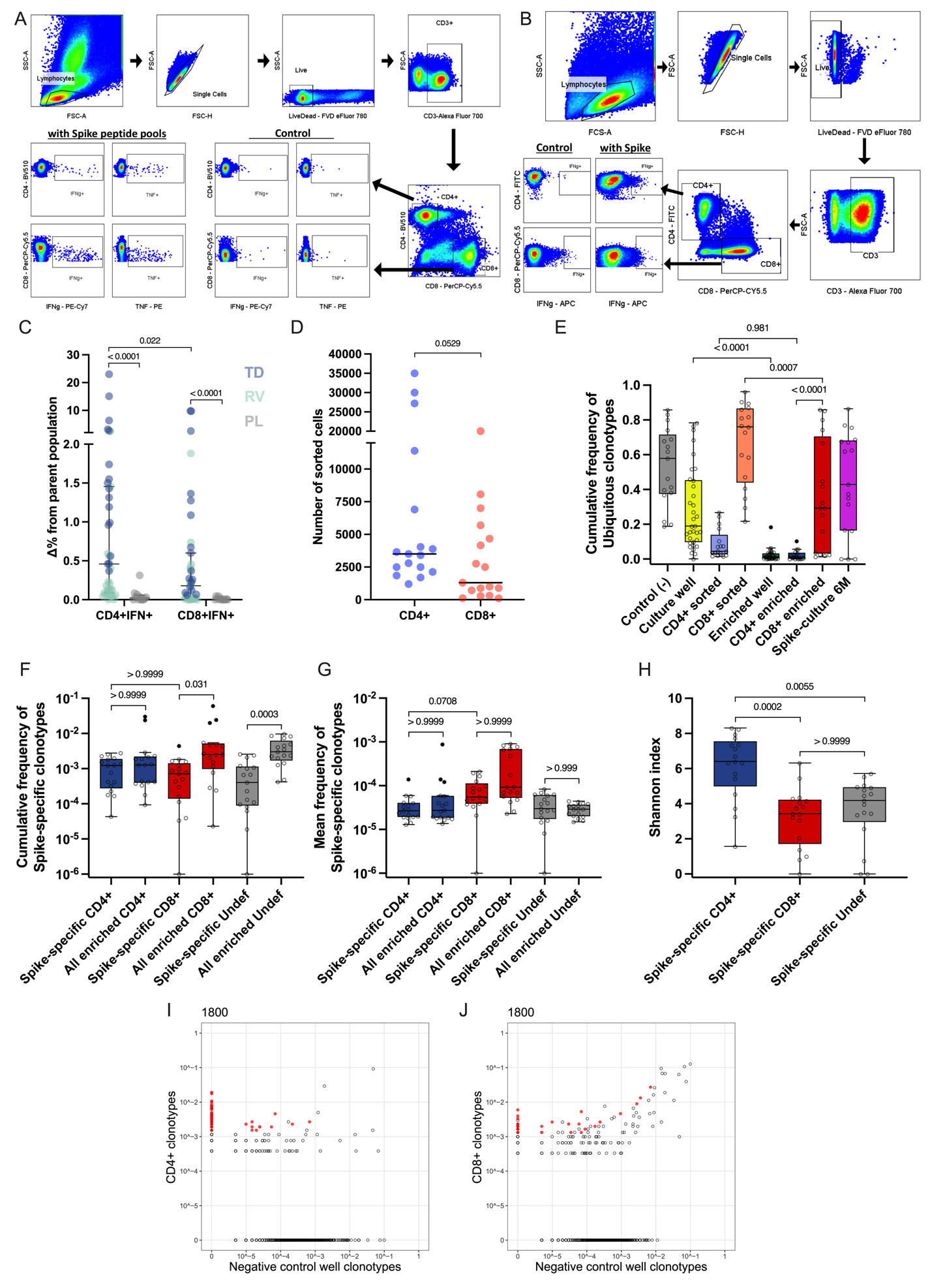


**Supplementary Figure 2. Gating strategy and analysis of groups of Spike-specific clonotypes.**

**A** - Gating strategy used to assess T cell activation after stimulation with a pool of peptides from the Spike protein by intracellular staining for the cytokines IFNg and TNF followed by flow cytometry.

**B** - Gating strategy used to sort IFNγ^+^ Spike stimulated CD4^+^ and CD8^+^ T cells after restimulation.

**C** - Percentage of IFNγ^+^ cells measured in TD, RV, and PL after restimulation of Spike-specific expansion (negative control subtracted).

**D** - Number of CD4^+^/ IFNγ^+^ and CD8^+^/ IFNγ^+^ cells sorted per donor in the TD group.

**E** - Cumulative frequency of ubiquitous clonotypes in different samples from vaccinated donors. Spike-expansion 6M - expanded Spike-specific culture from samples collected 6 months after vaccination.

**F and G** - Cumulative frequency (E) and mean frequency (F) of Spike-specific and all enriched clonotypes in total repertoire on day 14.

**H** - Shannon’s divercity index of Spike-specific clonotypes in CD4^+^, CD8^+^, and undefined fractions.

**I and J** - A representative enrichment plots for donor p1800, showing CD4^+^/IFNγ^+^ (H) and CD8^+^/IFNγ^+^ (I) sorted cells versus untreated control culture. Red dots represent clonotypes that are enriched.

In C-D, Mann-Whitney U-test was used to test statistical significance. For E-G, one-way analysis of variance (ANOVA) followed by Tukey's multiple comparison test was used. For H, significant Kruskal-Wallis test followed by Dunn's multiple comparison post hoc test was used. The median is shown on the graphs of independent experiments. PL - placebo group, TD - target donors, RV - remaining vaccinated group.

*
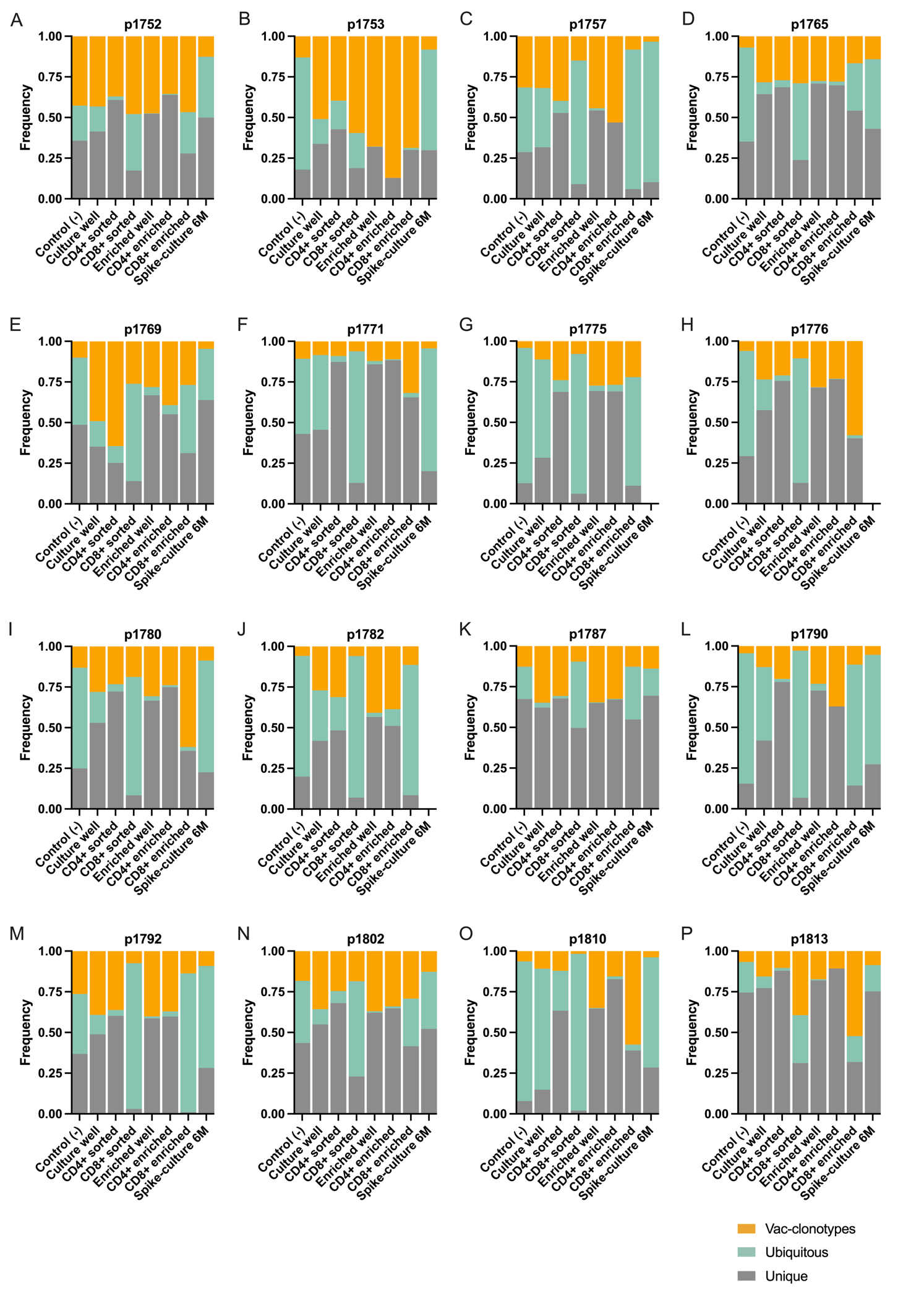
*

**Supplementary Figure 3. The distribution of frequencies of clonotype groups**.

**A-P** Frequency distribution of Vac-clonotypes (orange), ubiquitous (turquoise) or unique (grey) in different samples from all donors. Each graph shows the frequency distribution for each individual vaccine recipient included in the cohort. Each histogram bar shows the proportion of clonotype groups within each fraction sequenced and analyzed after T cell expansion with or without clonotype enrichment. Spike expansion 6M - expanded Spike-specific culture from samples collected 6 months after vaccination.


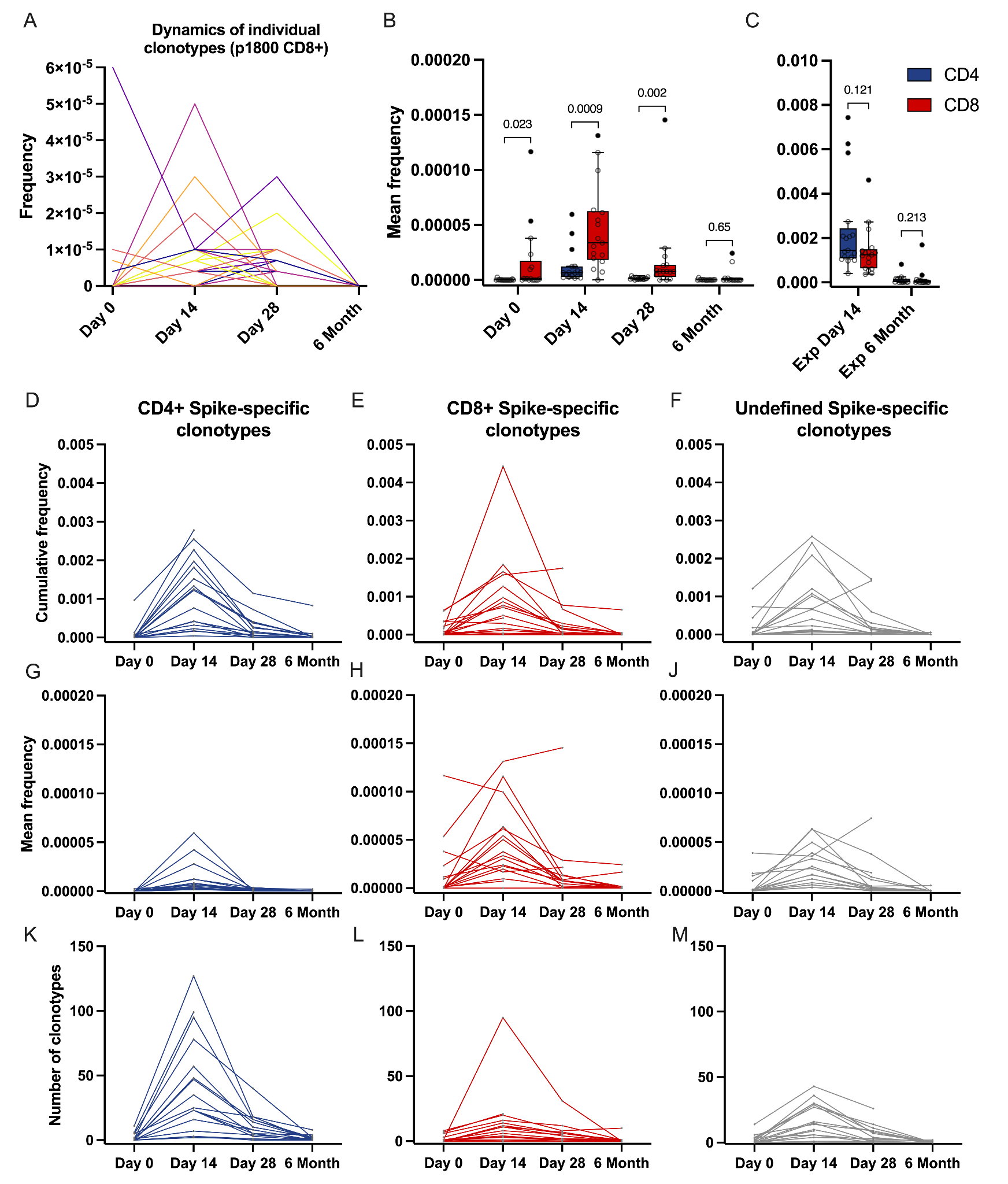


**Supplementary Figure 4. Tracking of Spike-specific clonotypes in each donor.**

**A** - Dynamic of the frequency of individual clonotypes for a representative donor. Each color reflects a single clonotype trajectory.

**B and C** - Dynamic of the mean frequency of CD4^+^ and CD8^+^ clonotypes, found in the total repertoire at 4 time points (B) or in the Spike–specific expansion at day 14 and 6 months (C).

**D-M** - Dynamics of cumulative clonotype frequency, mean clonotype frequency, and a number of clonotypes belonging to the CD4^+^, CD8^+^, and undefined Spike-specific subpopulations, found in the total repertoires at 4 time points. Lines correspond to individual donors. On A and B Mann-Whitney U-test was used to test statistical significance


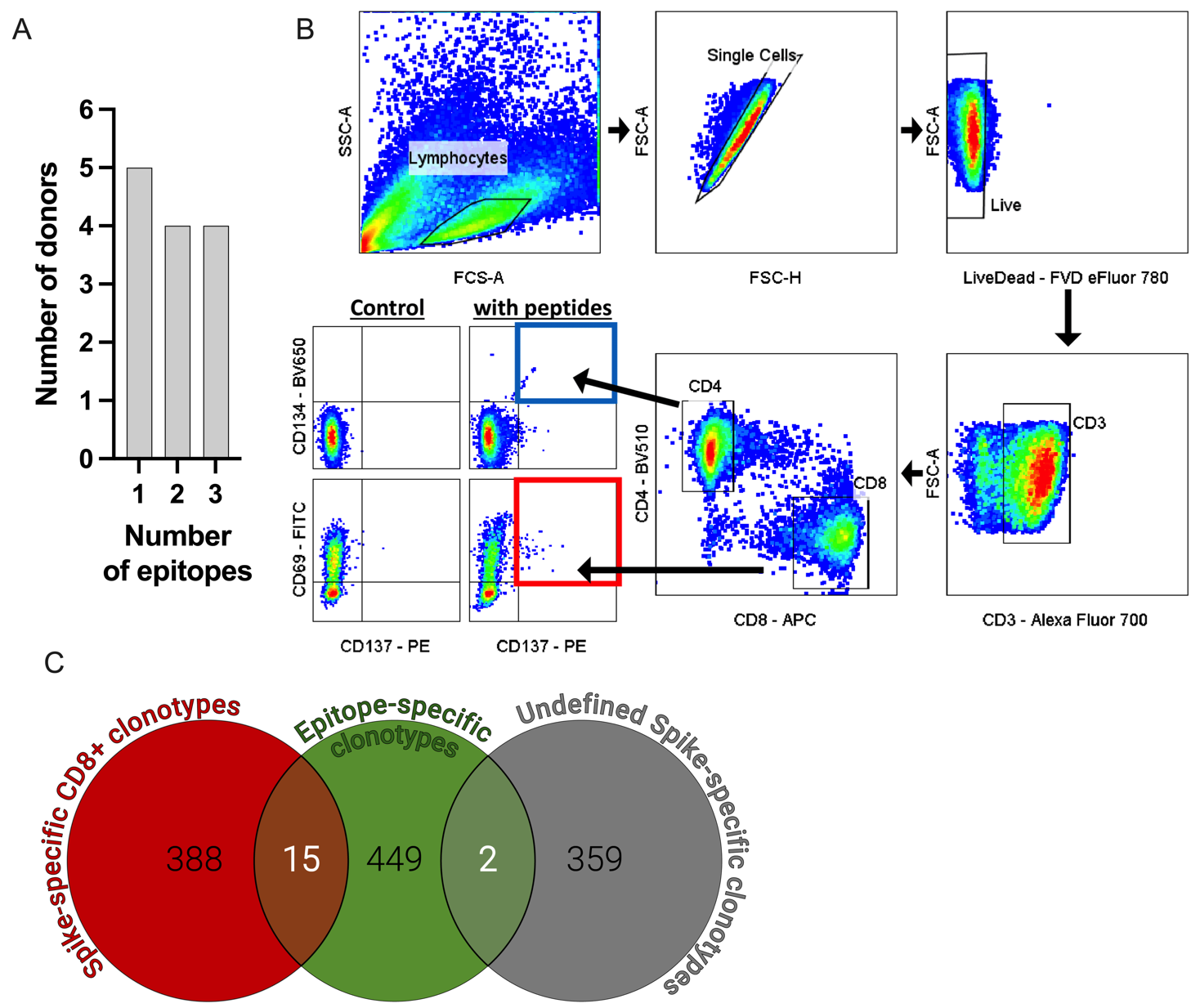


**Supplementary Figure 5.** AIM assay gating strategy and epitope distribution.

**A** - Number of donors responding to epitopes.

**B** - Gating strategy for AIM-sorting after the peptide-specific T cell expansion.

**C** - Venn diagram of epitope-specific (MHC class I) T cell clonotypes overlapping with Spike-specific CD8^+^ and undefined clonotypes for all donors.


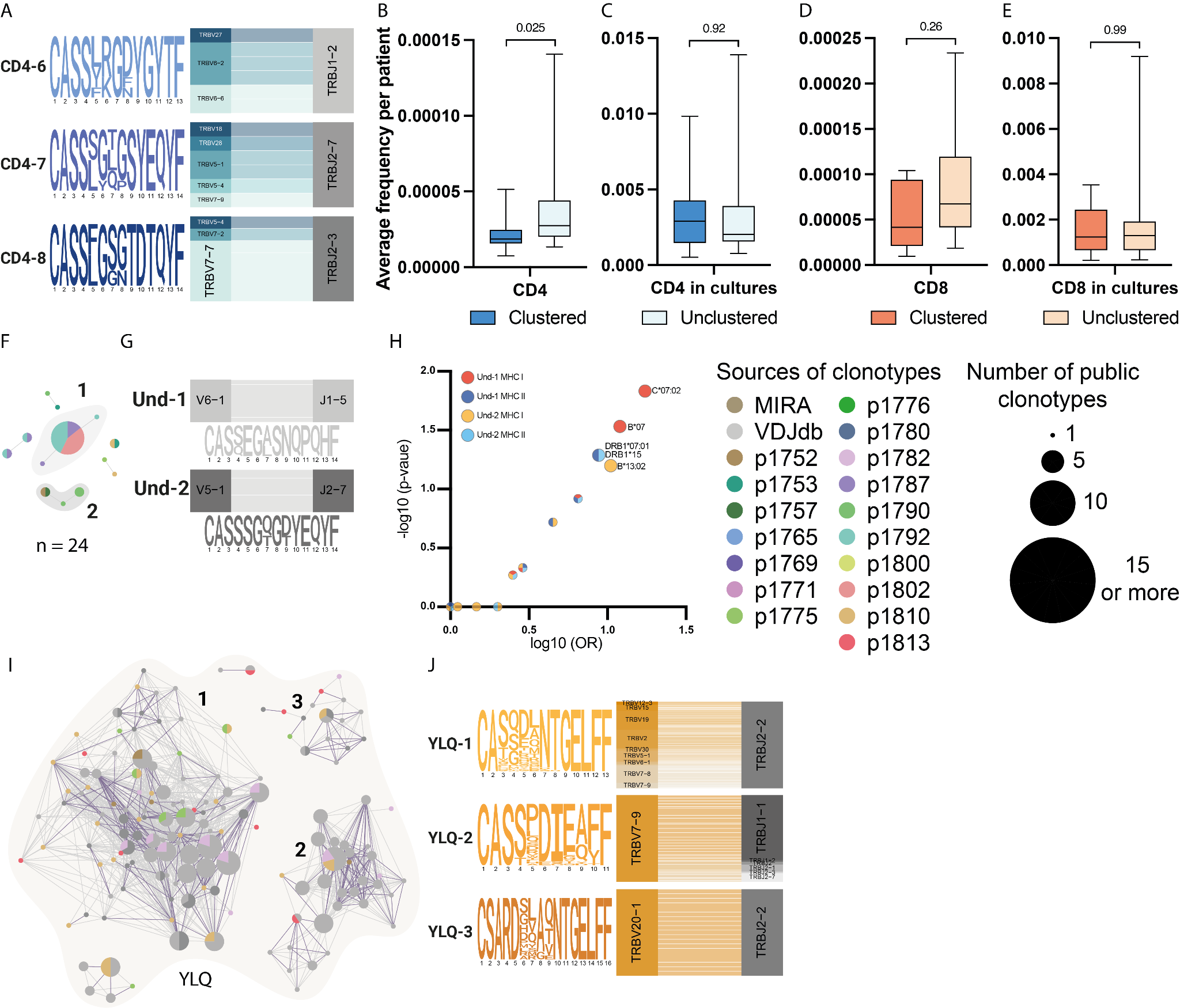


**Supplementary Figure 6**. **Similarity of epitope-specific clonotypes.**

**A** - Position-weight matrices for CDR3 and V-J-genes usage (Sankey plots) for СD4-6, -7, and -8 clusters from Fig 5A

**B - E** - Average frequency of all clustered and unclustered CD4^+^ (B and C) and CD8^+^ (D and E) Spike-specific clonotypes in the total repertoire of the 14th day.

**F** - Cluster of CDR3-regions of 24 undefined Spike-specific clonotypes with Hamming distance =2. Unclustered clonotypes are not shown. The size of the nodes in the legend reflects the number of identical (public) sequences, the color indicates the donors. Clusters of interest are named and highlighted.

**G** - Position-weight matrices for CDR3 and V-J-genes usage (Sankey plots). Cluster numbers correspond to the numbers shown in (G)

**H** - Volcano plot showing fold of each HLA in each cluster. Clusters are colored. Axes indicate the decimal logarithm of the clustering odds ratio versus the negative decimal logarithm of the p-value (Fisher’s exact test).

**I** - Clustering of YLQ-specific clonotypes with annotated sequences from databases.

**J** - CDR3 logos and V-J-genes usage (Sankey plots) for YLQ-specific clusters.

On B-F Mann-Whitney U-test was used to test statistical significance.
